# Supplementary material for: Exceptional water production yield enabled by batch-processed portable water harvester in semi-arid climate
Source: Nat Commun. 2022 Sep 15;13:5406. doi: 10.1038/s41467-022-33062-w (PMC9478113; doi:10.1038/s41467-022-33062-w)
Supplement: Supplementary file 1 — Supplementary Information [file 41467_2022_33062_MOESM1_ESM.pdf]

Supplementary Information

Exceptional Water Production Yield Enabled by Batch-processed Portable Water Harvester in Semi-arid Climate

He Shan[1,2], Chunfeng Li[2], Zhihui Chen[1,2], Wenjun Ying[2], Primož Poredoš[1,2], Zhanyu Ye[1,2], Quanwen Pan[2,*], Jiayun Wang[2], Ruzhu Wang[1,2,*]

[1] Institute of Refrigeration and Cryogenics, Shanghai Jiao Tong University, Shanghai 200240, China

[2] Engineering Research Center of Solar Power & Refrigeration, MOE China, Shanghai 200240, China

Supplementary Section 1. Characterization of Materials

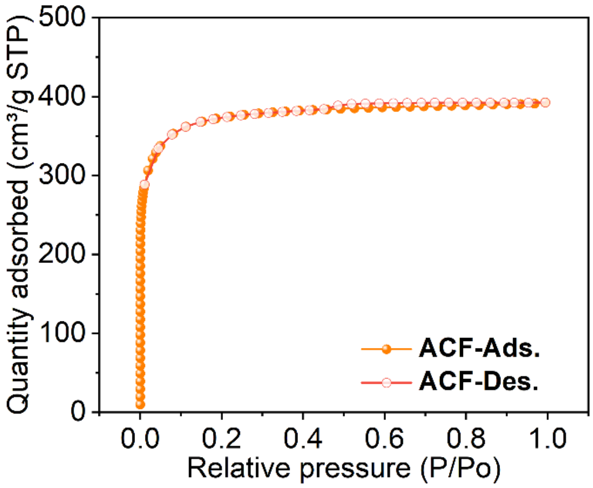

Supplementary Figure 1. Nitrogen adsorption and desorption isotherms of active carbon felt (ACF) matrix. The results demonstrate the domination of micropores, which can provide abundant pores for the attachment of LiCl crystals. Based on the results, the specific surface area of 1222.11 m^2/g was calculated by Brunauer-Emmett-Teller (BET) method.

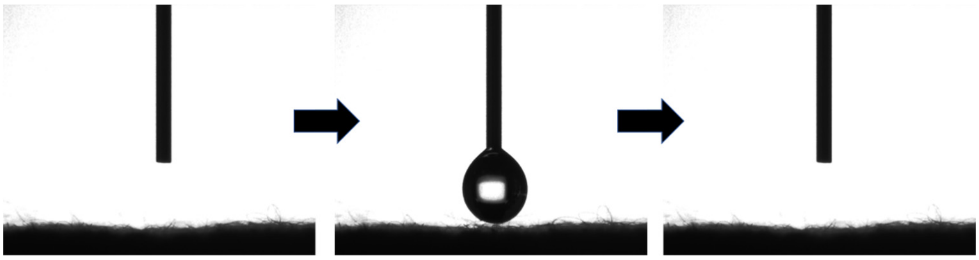

Supplementary Figure 2. Dynamic contact angle measurement of the porous matrix ACF. It demonstrates the strong hydrophilicity of the matrix, which could contain LiCl solution after the sorption process and partly avoid salt solution leakage.

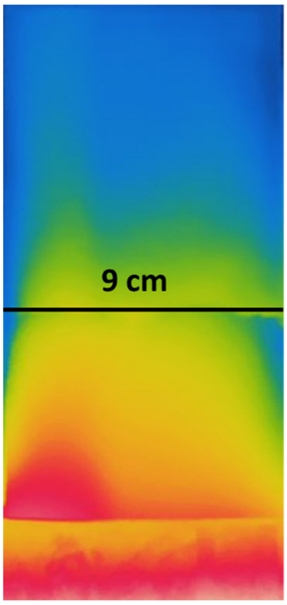

Supplementary Figure 3. Capillary force measurement. Infrared optical photos of absorbed water height inside the matrix. The height of the water inside the ACFF was more than 9 cm, indicating the strong capillary force property of the ACF matrix.

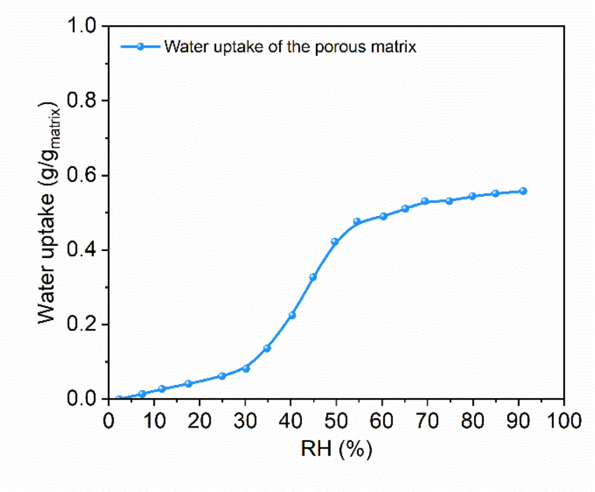

Supplementary Figure 4. Water vapor sorption isotherm of the porous matrix (ACF). It indicates the vapor sorption mechanisms of the micropore filling adsorption in micropores, the multilayer adsorption, and capillary condensation in mesopores. The relatively low water adsorption capacity (< 0.1 g g^{-1} below 30% RH) can hardly be suitable for water harvesting in arid climates.

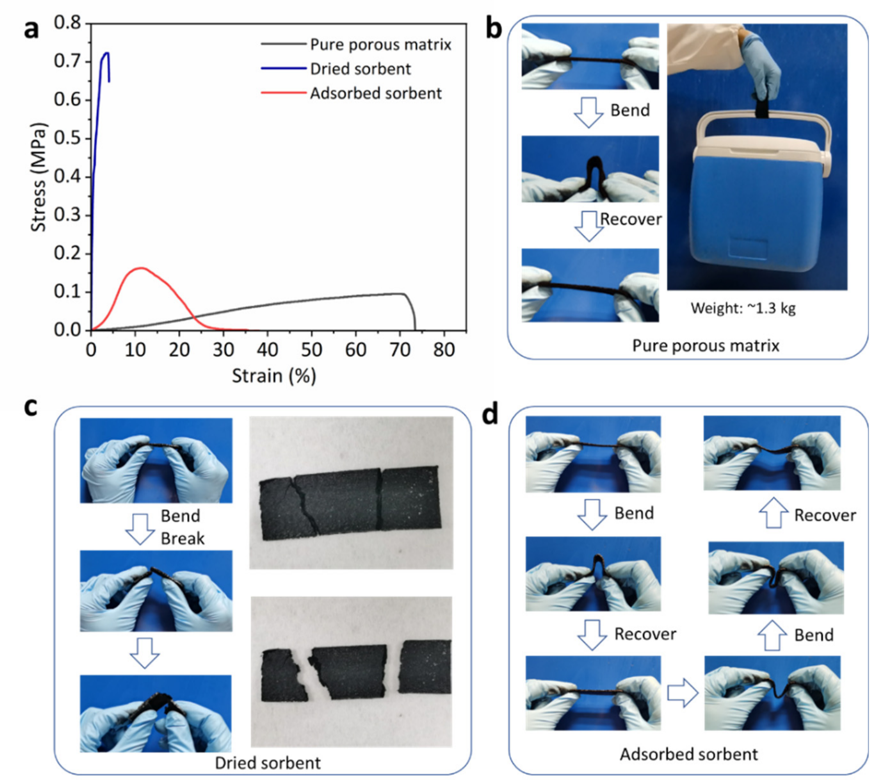

Supplementary Figure 5. Mechanical properties of the pure porous matrix, the dried as-prepared sorbent, and the sorbent captured water vapor at 70% RH. a) Stress-strain curves of the pure porous matrix, dried sorbent, and absorbed sorbent. The mechanical properties were measured by a dynamic mechanical analyzer with a loading rate of 20 mm/min. b) The photos that show the flexibility and the breaking strength of the matrix. c) The photos that show the non-stretchable behavior of the dried sorbent. d) The photos that show the recovered flexibility of the sorbent after capturing water vapor at 70% RH.

The stress-strain curve shows that the pure porous matrix shows excellent flexibility but relatively low breaking strength (0.095 MPa), indicating an elastic increase of the load before reaching the maximum stress. After that, the load decreased slowly with the displacement, which may result from the fiber-bridging and sliding after debonding and pulling out of carbon fibers[1]. Besides, as the photograph showed (Supplementary Figure 5b), the matrix can be easily bent and restored to its original state, demonstrating the flexibility of the pure matrix. Besides, a 1.3 kg box could be easily lifted by a 2 cm wide carbon fiber matrix without damage. After the LiCl salt impregnation, the dried as-prepared sorbent that loaded ~90 wt% salt shows a high breaking strength (0.723 MPa), but demonstrates non-stretchable behavior, indicating a brittle failure mode. LiCl particles may act as an interface binder to transfer the load to the

whole composite material[2]. The dried sorbent can be easily broken (Supplementary Figure 5c). However, after sorption at 70% RH, the sorbent becomes softer, and its flexibility is partly recovered (Supplementary Figure 5d) because of the formation and filling of the high concentration LiCl solution, showing a pseudoplastic behavior and an increased toughness[1, 3].

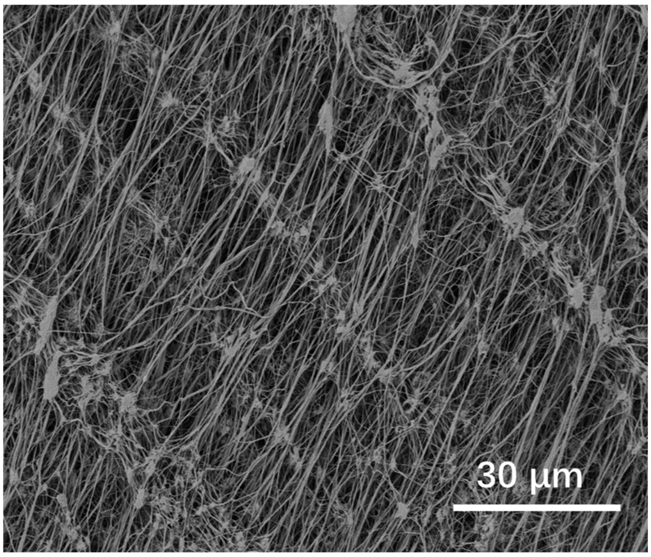

Supplementary Figure 6. SEM images of PTFE membranes with the pore size of 3 µm. It shows the highly parallel orientation of observed fibers and the formation of suitable pore size, caused by the fabrication process of PTFE particle stretching.

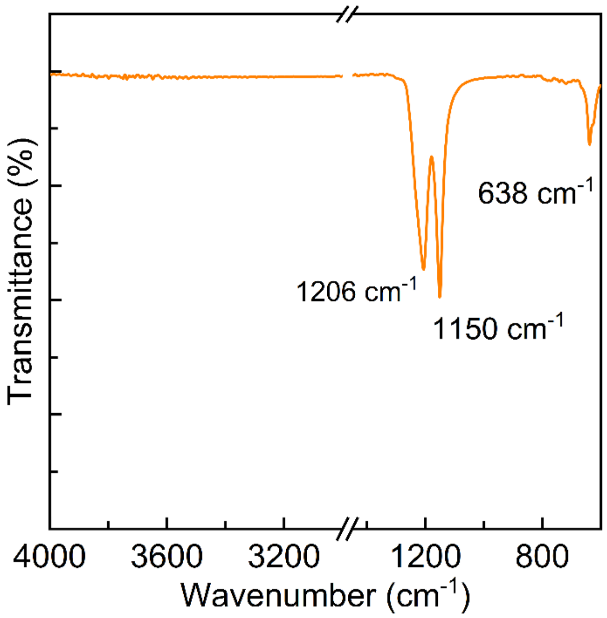

Supplementary Figure 7. ATR-FTIR spectrum of the PTFE membrane.

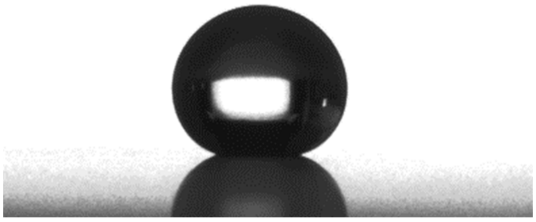

Supplementary Figure 8. Measured contact angle (~170°) of the PTFE membrane.

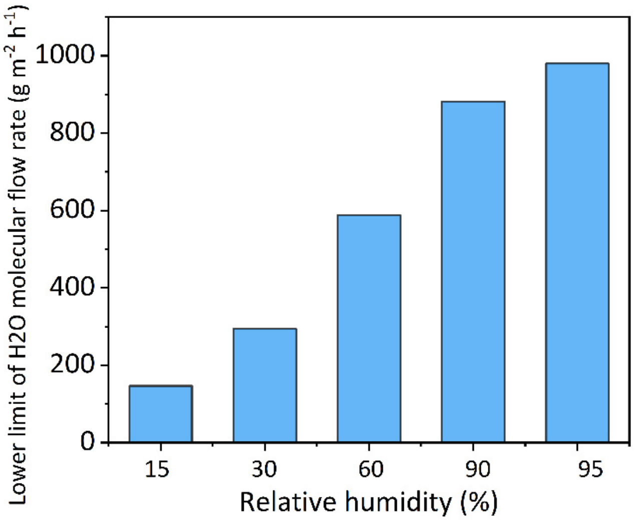

Supplementary Figure 9. The lower limit of the water molecular permeability at 15%, 30%, 60%, 90% and 95% RH, and 15 °C. It is calculated by the lower limit of the airflow rate (127.4 mL cm^{-2} min^{-1}) and the assumption of the uniform distribution of the carried water molecules by the air. The thickness, porosity, and pore size of the membrane are 20 μm, 0.87, and 3 μm, respectively. Theoretically, the mass transfer process relied on the vapor diffusion inside the membrane pores, which has been discussed extensively in the literature as a combined effect of Knudsen diffusion, molecular diffusion, and viscous (Poiseuille) flow[4, 5]. The dimensionless Knudsen number (Kn) can be a guideline to determine the domain mechanism, which is defined as the ratio of the mean free path of the gas *l* [m] to the pore size *r* [m], as follows:

$$\mathrm{Kn} = \frac{l}{2r}$$

The mean free path of the gas (water vapor) is the average traveled distance of the molecules between two collisions, given by:

$$l = \frac{k_B T}{\sqrt{2}\pi P \sigma^2}$$

where k_B is the Boltzmann constant 1.38×10^{-23} [m^2 kg s^{-2} K^{-1}], T the temperature [K], σ the collision diameter (0.2641 nm for water vapor), $\overline{P}$ the mean pressure in membrane pores [Pa]. The mean free path is estimated to be 0.133 μm in our study.

When the vapor diffusion through the membrane and the pore size is smaller than the water vapor mean free path (Kn > 1), the molecule-pore wall collisions dominate the mass transfer, which is called as Knudsen region. Oppositely, when pores are much bigger than the mean

free path (Kn < 0.01), the molecule-pore wall collisions are negligible[6]. Here in our study, the estimated Kn number is 0.04, which means the mechanism of vapor transfer inside the membrane belongs to the transitional region with 0.01 < Kn < 1[7].

Supplementary Section 2. Materials Optimization

The linear driving force (LDF) model was used to quantitively evaluate the sorption dynamics of the samples with different salt contents. LDF model assumes that the mass transfer resistances are lumped in a film inside the adsorbent particle[8, 9]. The heat transfer is assumed to be lumped in a film at the outside surface of the adsorbent. The diffusion rate into the adsorbent is essentially proportional to the difference between the equilibrium state and the current adsorbed state. The mass transfer governing equation is:

$$\frac{dC(t)}{dt} = k_L(C^* - C)$$

The boundary conditions and the equilibrium relationships are described as:

$$t = 0, C = C_0$$

$$t = \infty, C = C_\infty$$

$$C_0 = C_0^*(p_0, T_0)$$

$$C_\infty = C_\infty^*(p_\infty, T_0)$$

$$C^* = C^*(p_\infty, T)$$

C is the absorbate weight per unit weight of the absorbent at the time t. C_0 is the initial absorbate weight per unit weight, and C_∞ denotes the equilibrium water uptake of a sorbent with various salt content, which is summarized in Supplementary Table 1.

Supplementary Table 1. Equilibrium water uptake for samples with various salt content at 30 °C and 60% RH. Note that the equilibrium water uptake of the four samples (Li-SHC-40, 70, 80, 90) was directly recorded at 12 hours, as shown in Fig. 1e. Especially, Li-SHC-95 did not reach equilibrium at 12 hours due to the slow sorption dynamic. The equilibrium water uptake of Li-SHC-95 was different from this value at 12 hours.

Sample	Equilibrium water uptake at 30 °C, 60% RH
Li-SHC-40	1.47 g/g
Li-SHC-70	2.31 g/g
Li-SHC-80	2.64 g/g
Li-SHC-90	2.93 g/g

Li-SHC-95	3.10 g/g

In our cases, the temperature and pressure of the adsorbent are assumed to be uniform and constant due to the relatively slow sorption process. Hence, $T = T_0 = T_\infty$, and $p = p_0 = p_\infty$. Besides, the initial water uptake is 0 ($C_0 = 0$) because sorbents are put into an oven at the temperature of 120 ^{o}C for the dehydration process. Because of the uniformity of sorbents, the equilibrium sorption capacities of all samples can be identical. Hence, the LDF model can be expressed as:

$$C = C_\infty(1 - \exp(-k_L t))$$

where k_L is the sorption rate coefficient that can be used to evaluate the sorption kinetics of different samples.

In the above equation, the sorption rate coefficient k_{LDF} can be used to evaluate the sorption dynamics. We fitted the sorption dynamic curves of Li-SHC-40, 70, 80, 90 and obtained the rate coefficient, as shown in Supplementary Table 2 and Supplementary Figure 10.

The linear driving force (LDF) model assumes isothermal conditions, which is suitable for the conditions without significant temperature increase caused by sorption heat[10]. The inclusion of the heat balance to account for non-isothermal kinetics provides a more accurate process description but imposes a significant increase in complexity. For our 12-hour sorption cases, the LDF model provides a mathematically simple but adequate description of the dynamic sorption process, because temperature measurements showed no significant increase during the experiments, which resulted from low layer thicknesses and constant temperature being maintained in the environmental chamber[11]. Also, the empirical fitting diffusion parameter that was obtained directly from the uptake data is illustrated as a sensitive parameter in describing the properties of vapor transfer and sorption kinetics[8]. In addition to the LDF model, the kinetic models for hydrogel-based hygroscopic materials and powder sorbent layers were discussed, which considered the volumetric expansion of hydrogel and the porosity of powders, providing frameworks to model the sorption and desorption processes in hygroscopic materials[11, 12].

Supplementary Table 2. Sorption rate coefficients for samples with different salt contents.

Sample	Sorption rate coefficient k_L (s^{-1})
Li-SHC-40	2.07 x 10^{-3}
Li-SHC-70	6.86 x 10^{-4}
Li-SHC-80	4.50 x 10^{-4}
Li-SHC-90	2.40 x 10^{-4}
Li-SHC-95	1.07 x 10^{-4}

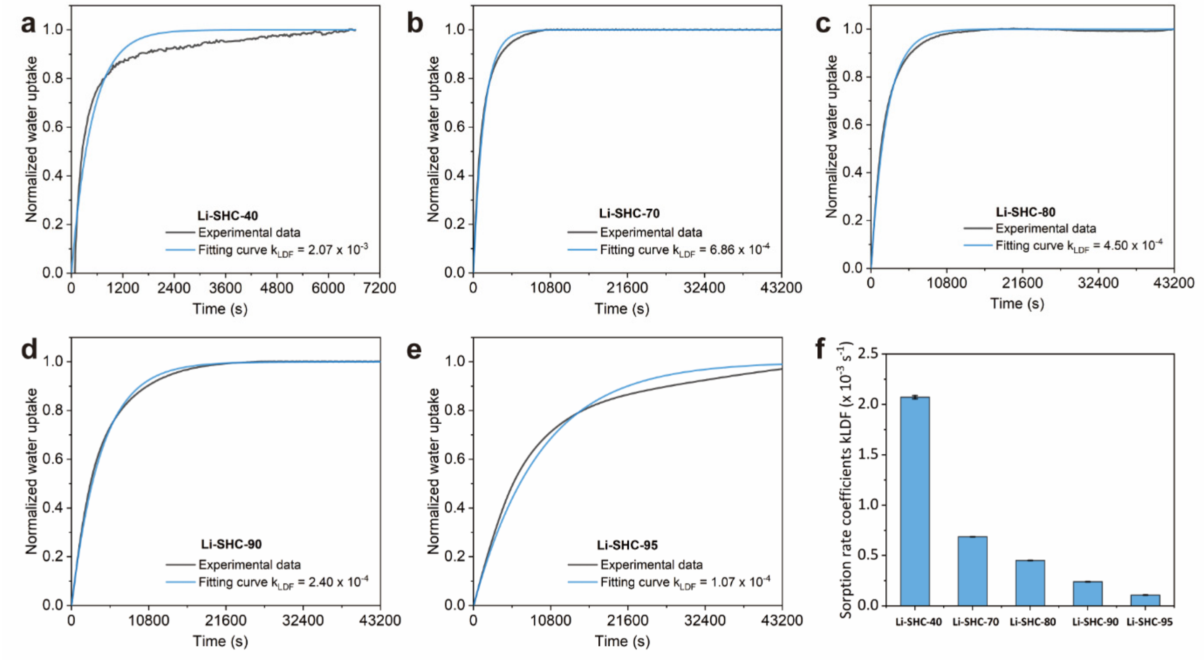

Supplementary Figure 10. Experimental dynamic sorption curves and LDF model sorption curves of samples with the salt content of a 40 wt%, b 70 wt%, c 80 wt%, d 90 wt%, e 95 wt%, and f the summary of the LDF coefficient.

The largest and smallest k_{LDF} obtained in the samples with highest and lowest salt content, respectively, indicates a strong positive correlation between salt content and sorption rate. Intuitively, as demonstrated in Fig. 1e, the equilibrium sorption duration increased from ~4 hours to over 12 hours with the salt content increased from 40 to 95 wt%. This variation of dynamic sorption characteristic owes to the mass transfer enhancement effect, provided by the matrix, as it leverages the effect of additional pores and channels for increased sorption interfacial area. However, with the increased salt content, lithium chloride crystals are formed

not only on the fiber pores but also on the surface and between the gaps of fibers, which inevitably reduces the sorption dynamics. That is why we evaluated the equilibrium and dynamic sorption performance of sorbents with different salt contents and finally selected the sorbent with the salt content of 90 wt%, which shows the best tradeoff between the dynamic and equilibrium performance. Therefore, the active carbon fiber matrix forms the bulk sorbent (Li-SHC composite sorbent), which is easy to use in the AWH device. Also, it avoids the direct utilization of the liquid absorbent (LiCl solution). More importantly, the provided reaction surface and vapor transport channels of the composite solid sorbent can accelerate the sorption process, obtaining a faster water uptake dynamic.

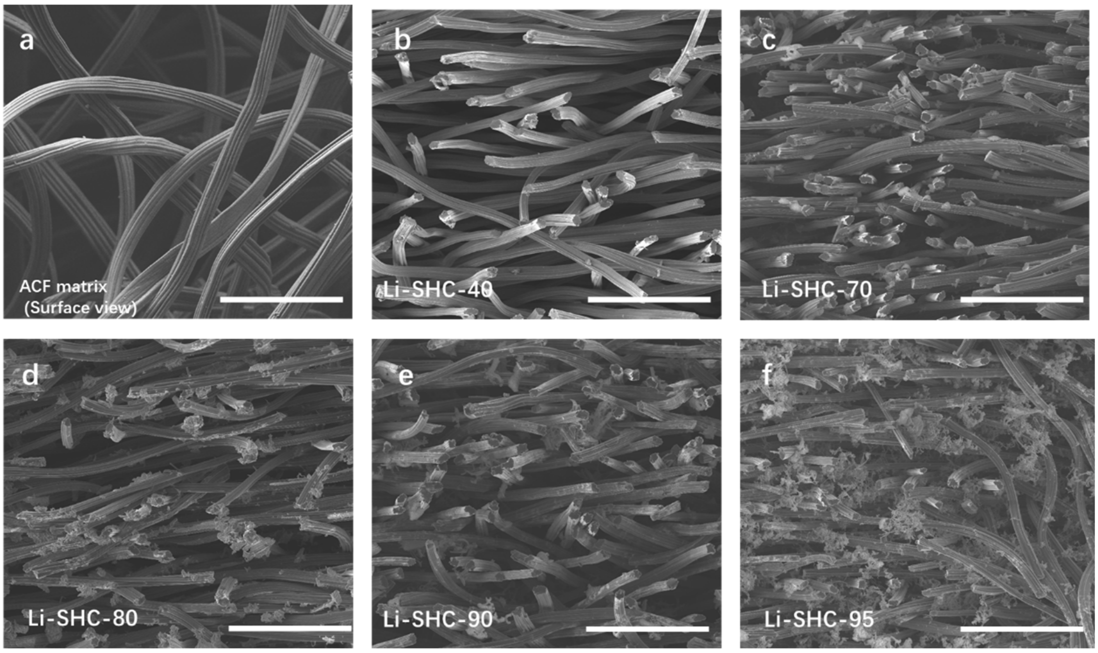

Supplementary Figure 11. Surface morphologies of **a** pure ACF matrix, **b** Li-SHC-40, **c** Li-SHC-70, **d** Li-SHC-80, **e** Li-SHC-90, **f** Li-SHC-95.

Supplementary Section 3. Sorption mechanisms

The equilibrium water uptake of the sorbent is controlled by the sorption characteristics of LiCl and its content. Hence, the sorbent Li-SHC shows multi-step water sorption mechanisms during the sorption process, which are chemisorption, deliquescence, and salt solution absorption. Based on this, we theoretically calculated the equilibrium water uptake and the sorption capacity in each step of the sorbent Li-SHC according to the sorption capacity of LiCl and its content.

The first step is the chemisorption of LiCl when the anhydrous LiCl crystals inside the composite sorbent capture water molecules and form hydrous salt ($LiCl\cdot nH_2O$, n=1,2,3,5). The value n is determined by the adsorption temperature. The phase diagram of LiCl and H_2O describe the crystallization lines of hydrous salts at different temperature. The crystallization line is described by the following equations, which are fitted according to the tested properties of the LiCl solution:

$$\theta = \sum_{i=0}^{2} A_i \xi^i$$

$$\theta \stackrel{\text{def}}{=} \frac{T}{T_{c,H_2O}}$$

where ξ is the mass fraction of the salt solution, T_{c,H_2O} is the critical temperature of water. The parameters A_i for different temperature and LiCl fraction ranges are included in Supplementary Table 3.

Supplementary Table 3. Parameters for the LiCl – H_2O crystallization line and phase diagram

Temperature range (°C)	LiCl fraction range	Crystal form	A_0	A_1	A_2
-75.5 to -68.2	0.253 to 0.287	$LiCl\cdot 5H_2O$	0.422088	-0.09041	-2.93635
-68.2 to -19.9	0.287 to 0.369	$LiCl\cdot 3H_2O$	-0.00534	2.01589	-3.11459
-19.9 to 19.1	0.369 to 0.452	$LiCl\cdot 2H_2O$	-0.56036	4.72308	-5.81105
19.1 to 93.8	0.452 to 0.558	$LiCl\cdot H_2O$	-0.31522	2.88248	-2.62433

over 93.8	over 0.558	LiCl	-1.31231	6.17767	-5.03479

This step contributes 0.42-0.85 grams of total water uptake and depends on the temperature. Typically for the sorbent, such as Li-SHC-90 operating under the ambient temperature (20-45 °C), the monohydrate $LiCl{\cdot}H_2O$ is formed and contributes 0.38 g/g water uptake in the case of the salt content of 90 wt%. Subsequently, the monohydrate salt crystals deliquescence and dissolve in the adsorbed water. The concentrated solution further absorbs water vapor and becomes dilute until the vapor pressure of the solution P_{sol} is in equilibrium with the external vapor pressure in air (i.e., relative humidity). The equilibrium mass fraction ξ of the LiCl solution relates to the overall water uptake w, given by:

$$w_{LiCl} = \frac{1-\xi}{\xi}$$

The equilibrium mass fraction was determined by the ambient temperature and relative humidity, which is followed by the following equations:

$$\frac{P_{sol}}{P_{water}} = C(A + B\theta)$$

$$A = 2 - \left(1 + \left(\frac{\xi}{\pi_0}\right)^{\pi_1}\right)^{\pi_2}$$

$$B = \left(1 + \left(\frac{\xi}{\pi_3}\right)^{\pi_4}\right)^{\pi_5} - 1$$

$$C = 1 - \left(1 + \left(\frac{\xi}{\pi_6}\right)^{\pi_7}\right)^{\pi_8} - \pi_9 e^{-\frac{(\xi-0.1)^2}{0.005}}$$

The parameters of π_i in the above equations are listed in Supplementary Table 4.

Supplementary Table 4. Parameters for the vapor pressure equation

π_0	π_1	π_2	π_3	π_4	π_5	π_6	π_7	π_8	π_9
0.28	4.30	0.60	0.21	5.10	0.49	0.362	-4.75	-0.40	0.03

Finally, the sorption capacity of the sorbent with the salt content of x can be theoretically calculated as the following equation:

$$w_{sorbent} = x \cdot w_{LiCl}$$

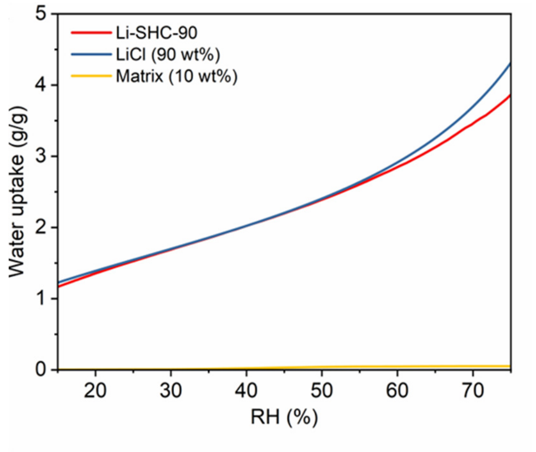

Supplementary Figure 12. Calculated isotherms of LiCl with the initial specific mass of 90 wt% and the experimental isotherms of Li-SHC-90 and the pure matrix

According to the above theoretical modes, the calculated isotherm of pure LiCl with the initial specific mass of 90 wt% is shown in Supplementary Figure 12. This theoretical isotherm shows high consistency with the experimental Li-SHC-90, indicating that isotherms of Li-SHC-90 sorbent and pure LiCl are essentially the same.

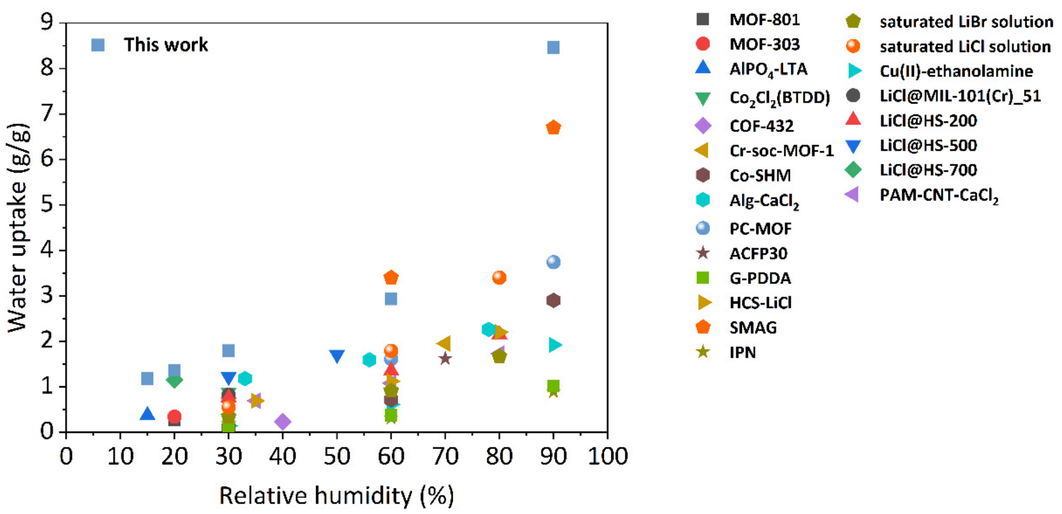

Supplementary Figure 13. Vapor sorption capacity comparisons of this work and previously reported materials.

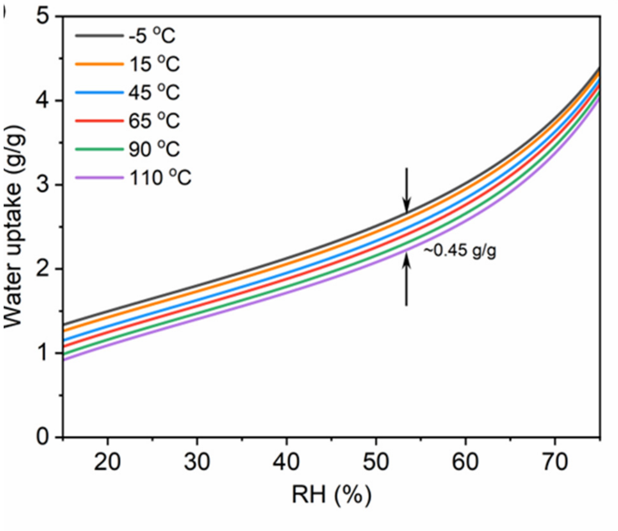

Supplementary Figure 14. Calculated theoretical isotherms of LiCl (90 wt%) at different temperatures. It demonstrates the insensitivity to temperature and sensitivity to relative humidity. This also shows the adaptability of sorbents to different climatic conditions (even lower than 0 °C).

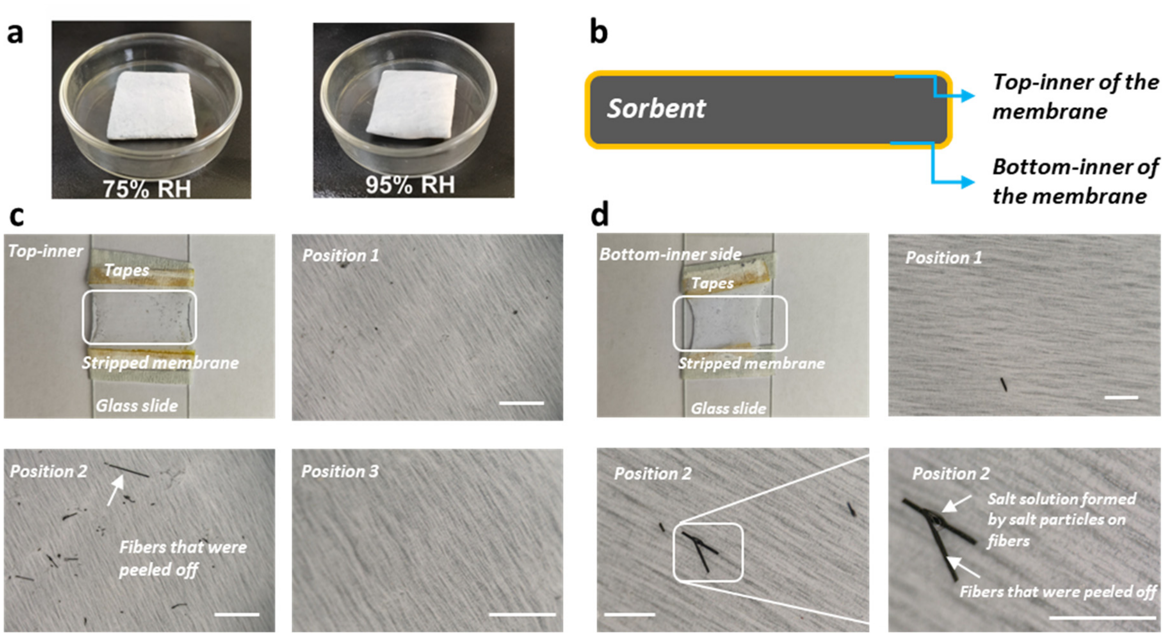

Supplementary Figure 15. Stability evaluation of Li-SHC.

a Photos of the sorbent Li-SHC after exposure to the high RH of 75% and 95% showing that the solution leakage was avoided by the membrane encapsulation. The risk of the salt solution was tested at high RH (75% RH and 95% RH), showing that no solution leakage was observed even at RH as high as 95%. It demonstrated the desirable stability of the sorbent Li-SHC, ensuring the reliability of the sorbents used in the batch-process mode. **b** Illustration of the positions of the stripped membrane. **c** Images of the stripped top-inner membrane that were captured by the optical microscope. Scale bar: 500 μm **d** Images of the stripped bottom-inner membrane captured by the optical microscope. Scale bar: 200 μm

To observe the possible leakage or accumulation of LiCl particles and solution more directly, we opened the encapsulation and stripped the membrane to show its state after 180-hour sorption-desorption cycles. To eliminate the effects of gravity, we stripped the membrane and split it into two pieces. As shown in Supplementary Figure 15b, one is the top-inner side of the membrane, and the other is the bottom-inner side of the membrane.

The stripped membranes were pasted onto the glass slides for further observation. No obvious salt particles were found on the surface of both the top-inner and bottom-inner sides of the stripped membrane. To show the state of the fibres more clearly and find out about the

existence of salt particles, the magnified images were further captured. Interestingly, no salt particles or droplets were found on the PTFE membrane but on the fibre fragments. It seems that the salt particles were carried down by the peeled fibres instead of the membranes. This result could be due to the significant differences of the hydrophilicity and hydrophobicity of the components. The PTFE membrane has a superhydrophobic characteristic (CA=170°, Supplementary Figure 8), and the fibre matrix is superhydrophilic (CA=0°, Supplementary Figure 2) with strong capillary force (Supplementary Figure 3), resulting in a significant difference in the energetic barrier of heterogeneous nucleation. [13] Therefore, the salt solution tends to be formed inside the hydrophilic matrix during the desorption and the salt crystallization. More importantly, owing to the aforementioned interactions between the solution, matrix and the membrane, the solution retains inside the matrix during the sorption-desorption cycling.

<u>Supplementary Section 4. Design of Water Harvester</u>

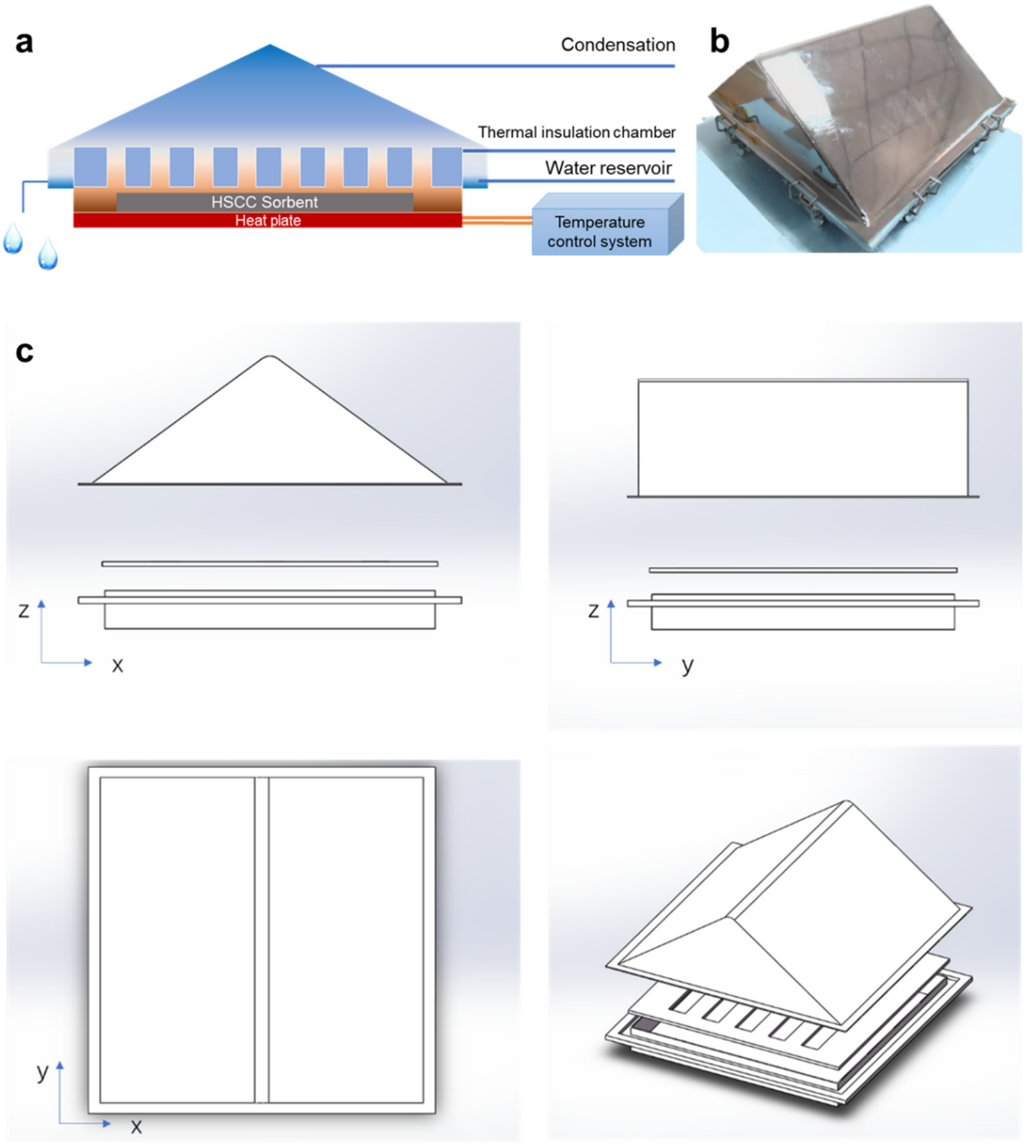

Supplementary Figure 16. The design of the water harvester. a structure diagram of a portable AWH device. **b** Photo of the water harvester **c** x-z, y-z (side)-views, y-x (top)-view and isometric view of the AWH device.

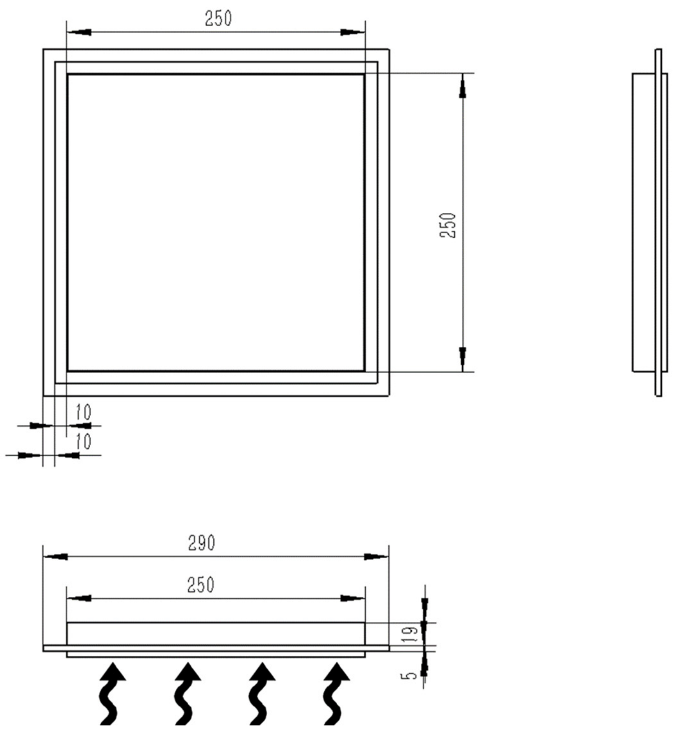

Supplementary Figure 17. Blueprints of the heating plate and its support structure.

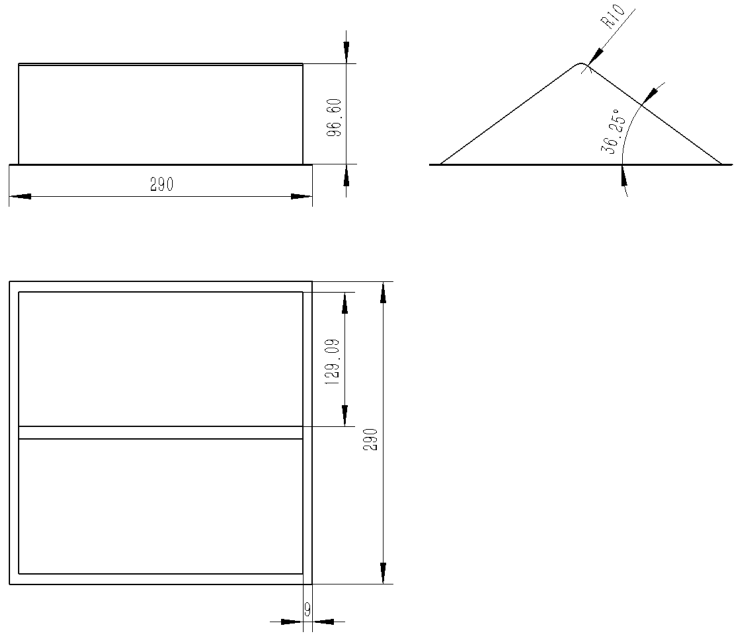

Supplementary Figure 18. Blueprints of the condensation cover.

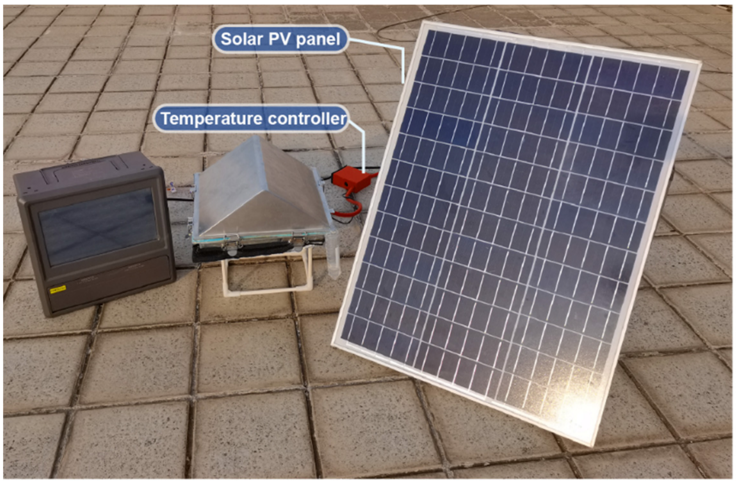

Supplementary Figure 19. Photo of the demonstration of the water harvester powered by the PV-battery system.

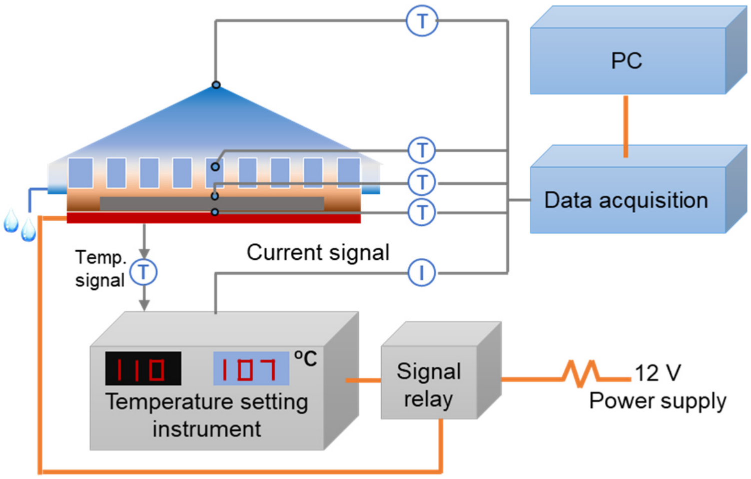

Supplementary Figure 20. Diagram of a temperature control system and data acquisition setup. The temperature control system consists of a temperature setting instrument, signal relay, and power supply. The temperature setting instrument is used to set the target temperature of the sorbent (DM30-An-A1, CW Electronic Instrument), and then it transfers this setting signal to the relay. A thermocouple is adhered to the surface of the heating plate to measure its temperature. Comparing the difference between the target temperature and measured temperature, the operation of the heating plate is controlled by changing the state of the signal relay. The control system uses the PID feedback control method to accurately control the heating temperature, and its fluctuation is within 2.5°C.

As for the data acquisition system, an ultra-thin K-type thermocouple with a diameter of 0.1 mm (temperature range -20 - 200ºC, accuracy ±1°C) is used to measure temperatures at the following measure points: the upper surface of a heating plate, the inner surface of a condensation part, the central point of sorbents, and along the vapor flowing path. The temperature signals were recorded by the data collector and processed by PC.

Supplementary Section 5. Optimization of Water Harvester

COMSOL simulation

The geometrical model of the water harvester for simulation is shown in Supplementary Figure 21. The temperature and velocity fields of the AWH device were simulated by COMSOL software at the ambient temperature and relative humidity of 25°C and 50% RH, respectively. The following assumptions and boundary conditions are applied for the model:

(1) The moist air in the sealed device was influenced by gravity.

(2) The airflow within the cavity is assumed to be laminar due to the relatively small height of the device.

(3) The moist air is assumed as a weakly compressible fluid because of the large temperature and density changes.

(4) No-slip boundary condition is applied.

The governing equations for moist air are as follows:

Continuity equation:

$$\frac{\partial \rho_a}{\partial t} + \nabla(\rho_a \boldsymbol{u}_a) = 0$$

Momentum equation:

$$\rho_a \left[\frac{\partial \boldsymbol{u}_a}{\partial t} + (\boldsymbol{u}_a \cdot \nabla)\boldsymbol{u}_a\right] = -\nabla P_a + \mu_a \nabla^2 \boldsymbol{u}_a + \rho_a \boldsymbol{g}$$

Energy equation:

$$\rho_a C_{p,a}\left(\frac{\partial T_a}{\partial t} + \boldsymbol{u}_a \cdot \nabla T_a\right) + \nabla \cdot (-\lambda_a \nabla T_a) = 0$$

in which, ρ_a is the density of moist air, kg/m^3; $\boldsymbol{u}_a$ is the velocity vector of moist air, m/s; T_a is the temperature of moist air, K; $\boldsymbol{g}$ is the gravity vector, 9.81 m/s^2 in the negative $\boldsymbol{y}$ direction; P_a is the pressure inside the chamber, Pa; λ_a is the thermal conductivity of moist air, W/(m·K); μ_a is the dynamic viscosity, Pa·s.

No moist air exists inside the walls of the heat insulation panels, as walls are not permeable for water vapor, hence the following energy balance was used:

$$\rho_w C_{p,w}\left(\frac{\partial T_w}{\partial t} + \boldsymbol{u}_w \cdot \nabla T_w\right) + \nabla \cdot (-\lambda_w \nabla T_w) = 0$$

The index w means the equation was applied only inside the walls of insulation panels.

Boundary conditions:

(1) Natural convection conditions in a large space are set for the condensation cover.

(2) The bottom heating plate is set to a constant temperature of 383.15 K.

(3) The initial temperature of the air is uniformly set as T_{amb} and the velocity is set to zero.

$t = 0$ for moist air,

$$\boldsymbol{u_a} = 0$$

$$T_a = T_{amb}$$

$y = 0$ for moist air,

$$T_a = T_w = 383.15 \text{ K}$$

For walls at $t = 0$,

$$T_w = T_{amb}$$

in which, T_{amb} is ambient temperature.

$$t > 0, y = 0,$$

$$T_w = 383.15 \text{ K}$$

$t > 0$, boundary conditions at wall surfaces in contact with the ambient:

$$-\boldsymbol{n} \cdot (-\lambda_a \nabla T_a) = h(T_{amb} - T_w)$$

$t > 0$, boundary conditions at wall surface in contact with the moist air:

$$-\boldsymbol{n} \cdot (-\lambda_a \nabla T_a) = h_a(T_a - T_w) + \varepsilon\sigma(T_{w'}^4 - T_w^4)$$

where natural convection heat transfer coefficient, $h = 10$ W/(m^{2}K)[14]; h_a is the heat transfer coefficient between the moist air and the wall, which is related to the velocity of the wet air near the wall[15].The temperature of the condensation cover obtained by experiment and simulation is shown in Supplementary Figure 22, confirming the accuracy of the simulation results.

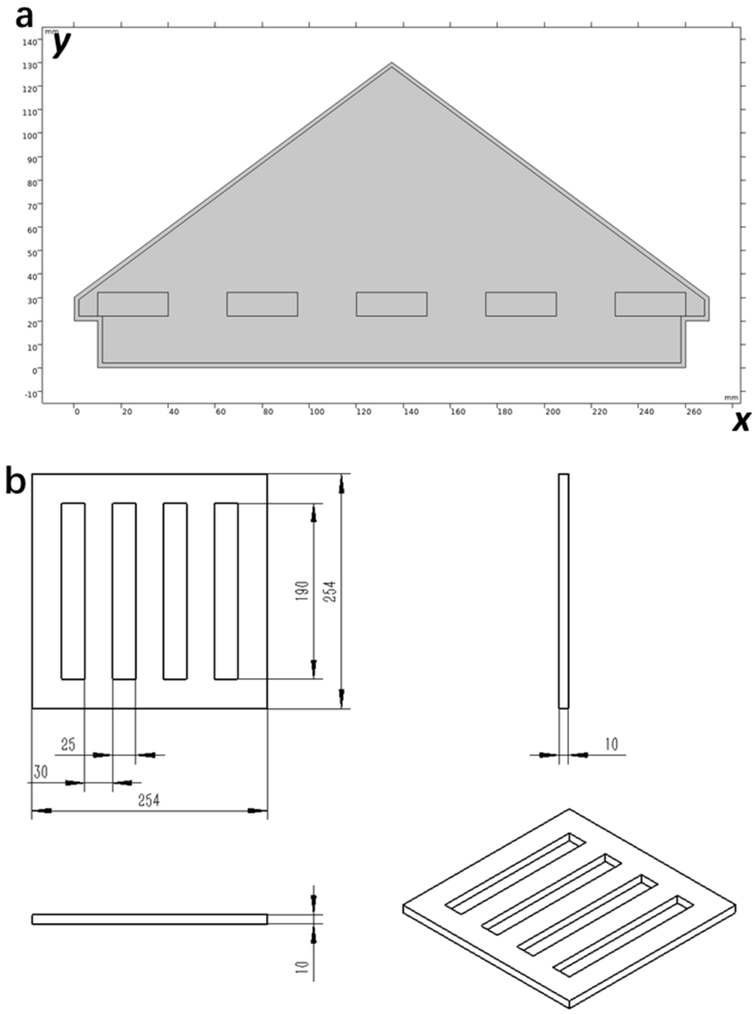

Supplementary Figure 21. **a** Geometrical model of AWH device for simulation **b** Blueprints of the insulation panel.

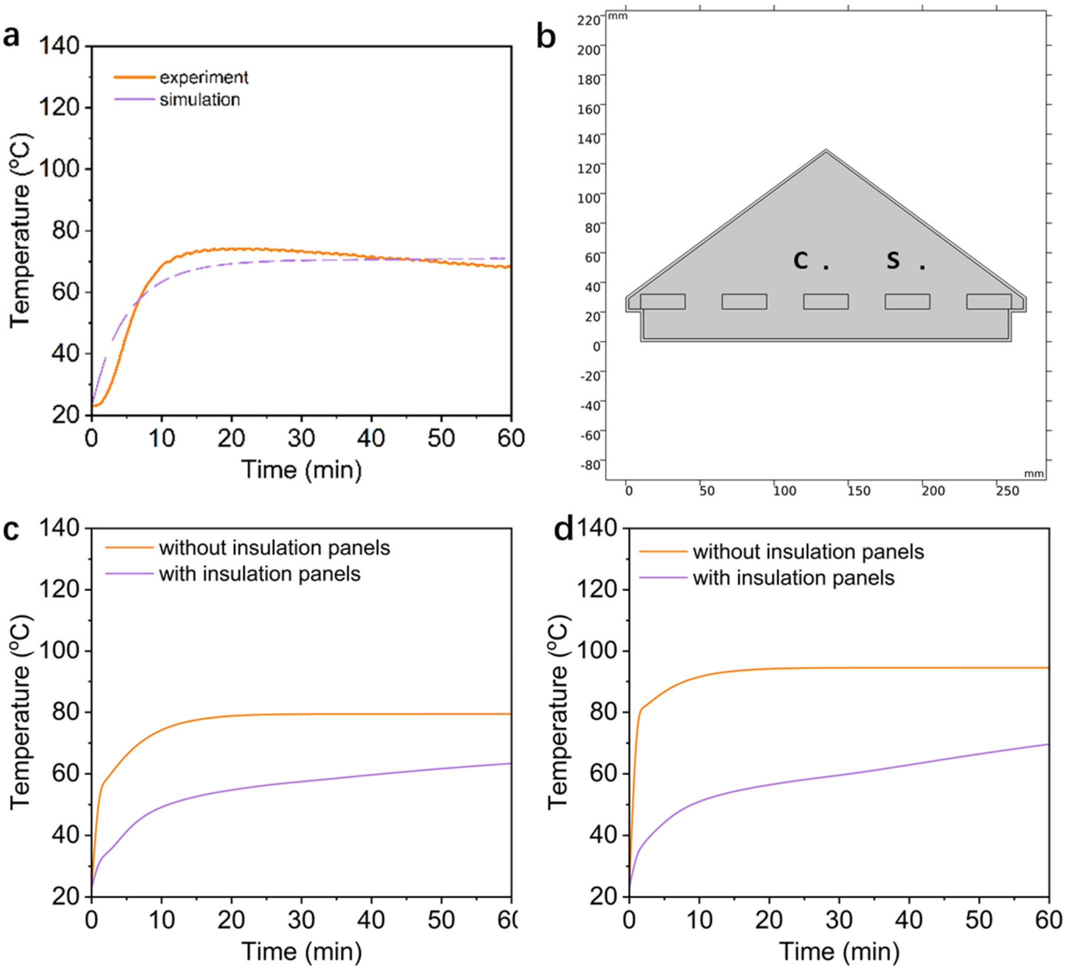

Supplementary Figure 22. a Temperature variation of simulated and experimental results. The temperature of the condensation cover obtained by experiment and simulation confirmed the accuracy of the simulation results. **b** Schematics showing the location of two characteristic points; **c** and **d** Temperature variations of characteristic points C and S with and without insulation panels. The simulation temperature variations of characteristic points (C (135, 50); S (200,50)) during the desorption process show the effect of increased temperature gradient by applying the insulation panel in two different locations.

Operation parameters optimization

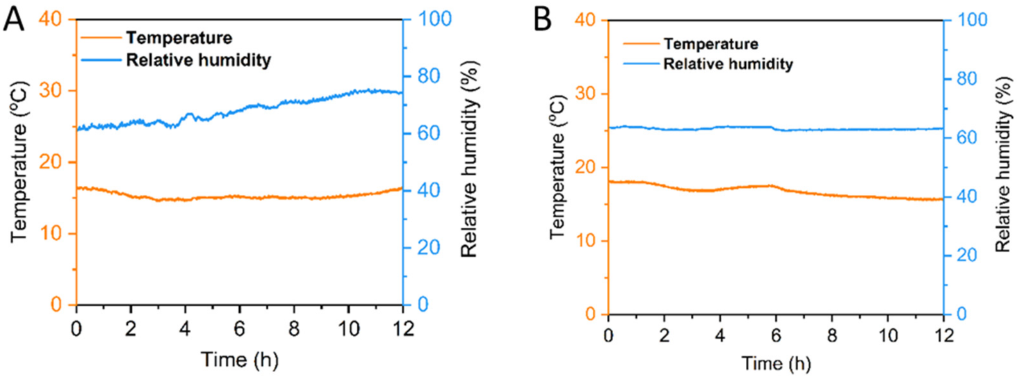

Supplementary Figure 23. Nighttime sorption conditions. The temperature and humidity variation on **a** Day 1, 21st September 2021, and **b** Day 2, 22nd September 2021. The experiments were conducted continuously for two days in Lanzhou, China from Sept. 21st to Sept. 22nd, 2021. It shows the typical temperature and relative humidity variations throughout the day during the experiment. The sorption process lasted for 12 hours, starting at 20:00 and finishing the next day at 8:00. It could be found that the RH range was 50%~75% and the average RH was 65% during the nighttime sorption process. Due to the increased solar radiation, the ambient temperature increased rapidly, and correspondingly, the relative humidity dropped to the minimum value.

The dry weights of sorbents were between 37.90-39.65 g, and the water uptake of sorbents was 1.83-2.19 g/g, which depended on the climate conditions (temperature, air velocity, humidity, etc.). This result is lower than the sorption capacity in the lab-scale condition, which is expected. The differences come from two main factors, which are the salt content and its corresponding uniformity within the sorbents and the practical sorption ambient conditions. Firstly, the scale-up synthesis of Li-SHC resulted in uneven salt distribution and various salt content throughout the sorbent. Secondly, the practical ambient conditions, including air flow, temperature, and local relative humidity, were highly fluctuating, rendering ambient conditions much different from ones obtained in the constant environmental chamber. More importantly, the airflow conditions in the ambient chamber were generally better than the real conditions in which the sorbent was placed in a box-shaped container.

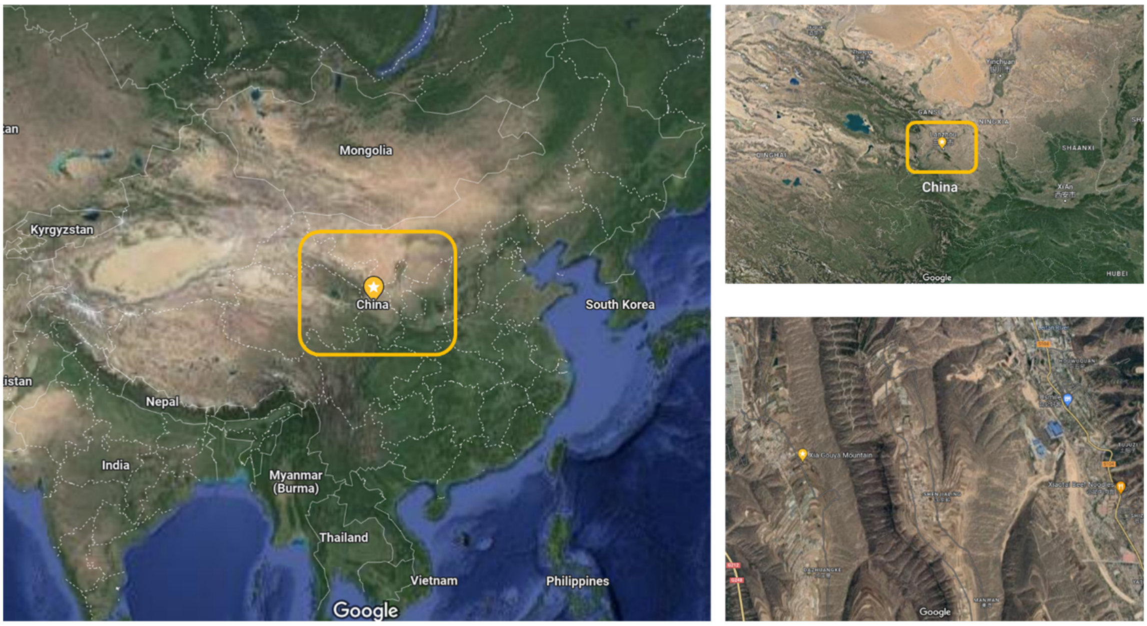

Supplementary Figure 24. Location of water harvester field tests (Xiagouya Mountain, Lanzhou, China, 36.017^{o} N, 103.784^{o} E. These maps were downloaded from Google Maps and used under principles of fair use.

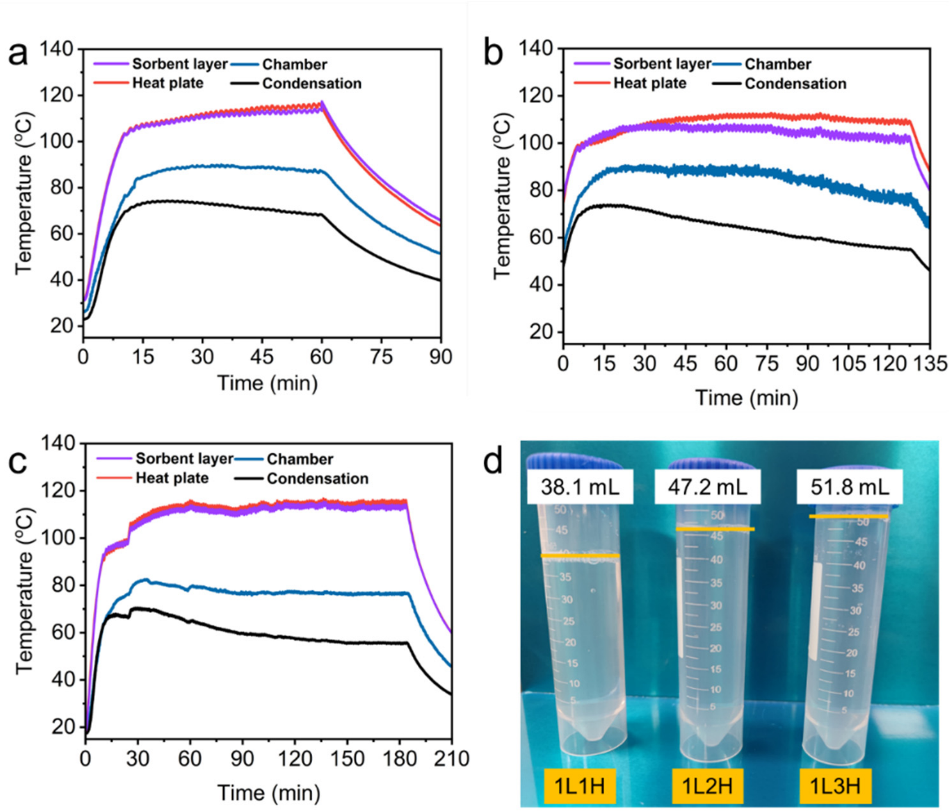

Supplementary Figure 25. Temperature variations for 1-layer experiment cases with different heating times. a 1 hour; b 2 hours; c 3 hours. d Digital photos of obtained water. L-layer(s); H-hour(s).

The heating temperature was set to 110°C. It took about 15 minutes to preheat the sorbent, as its temperature increased from the ambient temperature to the desorption temperature. Then, the temperature was stable during the whole experiment process due to the accurate operation of the temperature control system. The temperature difference between the heating plate and the sorbent was kept within 5°C, proving the sufficient heat transfer characteristics of one-layer sorbent. The condensation temperature was over 70°C during the first 30 minutes. Meanwhile, the sorbent released over 60 wt% of adsorbed water according to dynamic desorption curves. Then, the condensation temperature gradually dropped due to the reduced vapor amount and released latent heat of condensation. More importantly, a blinds-style high reflectivity and heat insulation chamber was designed to prevent the development of natural convection within the chamber and the direct thermal radiation between the condensation surface and sorbents. Therefore, a large temperature difference

between the condensation part and sorbents could be found and the minimum value was ~30°C, ensuring the vapor-liquid conversion effect.

The obtained water amounts of different heating times were shown in Supplementary Figure 25d, which were 38.1, 47.2, and 51.8 mL for 1L1H, 1L2H, and 1L3H configurations, respectively. Considering the desorption, condensation, and collection processes, the overall water yield rates were 31.5%, 39.1%, and 44.0%, respectively.

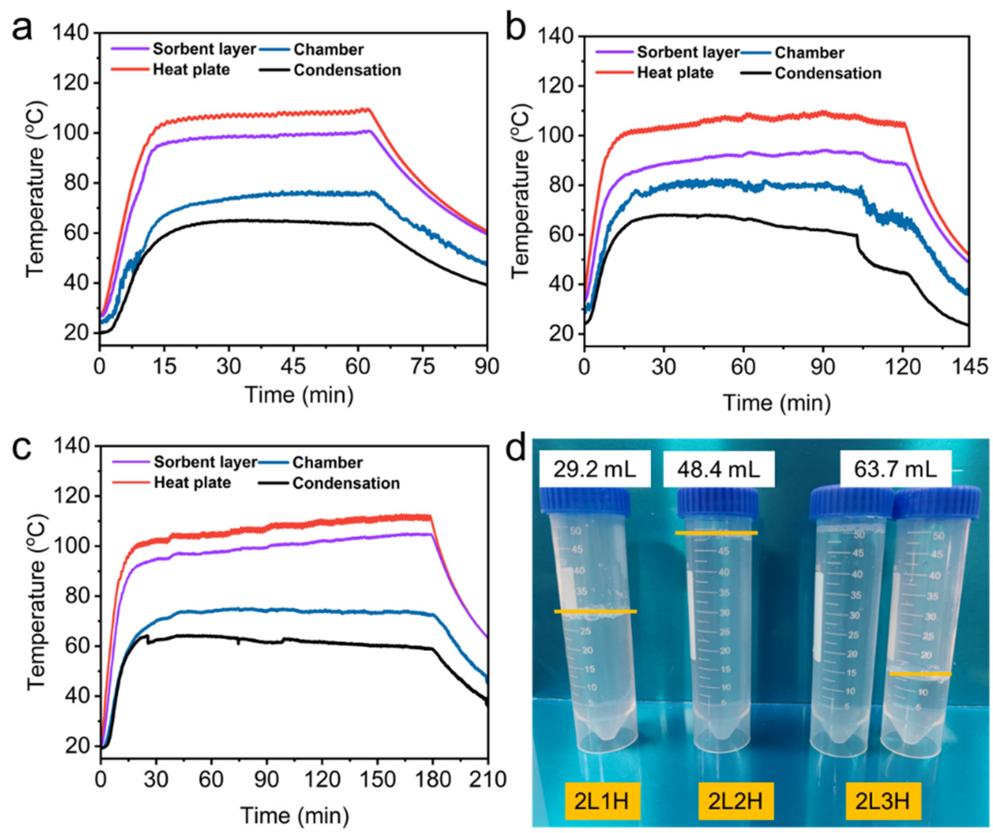

Supplementary Figure 26. Temperature variations for 2-layer experiment cases with different heating time a 1 hour; **b** 2 hours; **c** 3 hours. **d** Digital photos of obtained water. L-layer(s); H-hour(s).

As for two-layer cases, the average temperature difference between sorbents and heating plate was 8~15 °C, which was higher than one-layer cases due to the increased heat transfer resistance of a layer of sorbent and the air gap between sorbents. Other temperature variations of two-layer cases were similar to the one-layer cases. The obtained water of 2L1H, 2L2H, and 2L3H were 29.2 mL, 48.4 mL, and 63.7 mL, corresponding to the overall water yield

rate of 25.0%, 42.0%, and 57.0%.

Compared to the one-layer cases, the larger mass transfer resistance between the two layers and higher thermal capacity needs to be considered. Therefore, the water yield of the two-layer sorbent cases was lower than that of one-layer cases when the heating time was within 1 hour, but when the heating time was two hours or more, the sorption capacity of sorbents became the main factor affecting water yield.

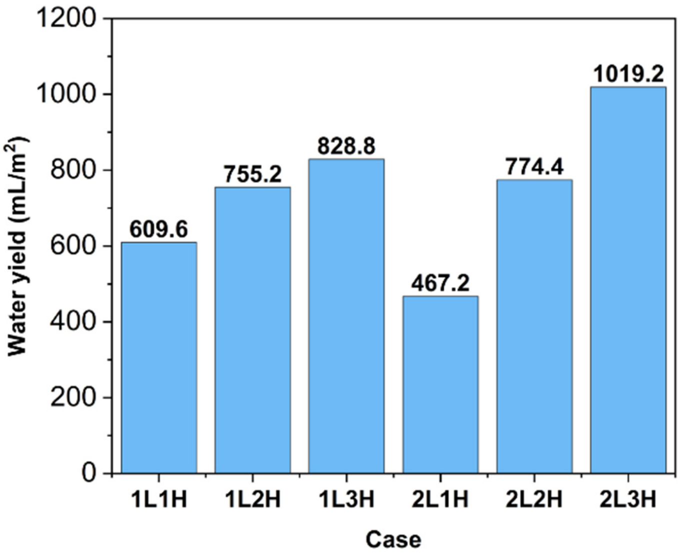

Supplementary Figure 27. Water yield performance evaluation.

The absolute amount of collected water increased with longer heating time because of higher desorption percentage regardless of one- or two- layer(s). As for the effect of sorbent layer number, the water yield of the two-layer sorbent cases was higher than that of single-layer cases, which is expected for one-hour desorption cases. The main reason behind this is the sensible heat demand of 2-layer sorbents was higher, and it took more time to reach the regeneration temperature, but after achieving a nearly steady-state, the sorption capacity becomes the main factor. The calculated water yield per hour and per unit of energy consumption (Fig. 3f) was analyzed in detail. The maximum water productivity per hour was 609.6 mL/(m^2·h), which was obtained in the 1L1H case due to the high desorption rate in the first hour. As for energy consumption, the maximum water yield per kilowatt-hour (5216.1 mL/(m^2·kWh) also occurred in the 1L1H case due to the limited heating time and its fast initial desorption rate.

The unit water yield decreased dramatically with the increased heating time due to the slower water release rate. It demonstrated only 339.7 mL/m^2·hour by 2L3H case even though the maximum absolute water yield of 63.7 mL was obtained. Especially for the 1L3H case, the water yield per kilowatt-hour reached only 3274.4 mL/m^2·hour. It was proven that when the heating time increased from two to three hours, more input energy cannot be used for generating larger amounts of vapor because at this time the sorbent already reached the desorption equilibrium.

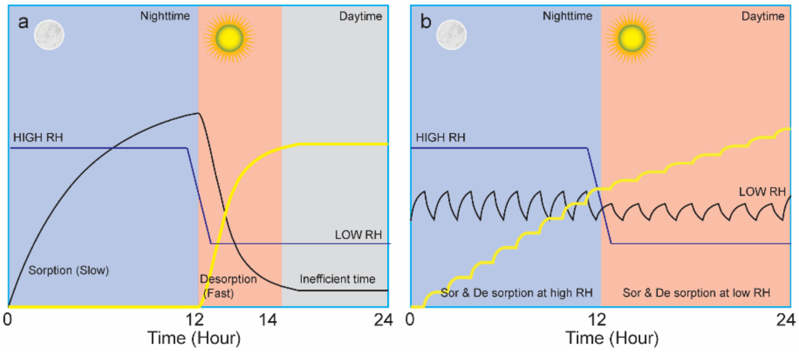

Supplementary Fig. 28 Illustration of different operation strategies used for atmospheric water harvesting.

a Traditional single water capture-release cycle in a diurnal cycle. Blue line – RH variation. Black line – Water sorption amount. Yellow line – Water production over the whole day. As the desorption rate is much higher than the adsorption rate, much of the daytime (around 10 hours) is wasted.

b Semi-continuous water capture-release mode. Blue line – RH variation. Black line – Water sorption amount. Yellow line – Water production over the whole day. The temperature variation caused by the switch of sorption and desorption processes causes waste of energy, meanwhile, the low RH during the daytime causes inefficient water uptake.

Supplementary Section 6. Cost Analysis of Water Harvester and Sorbent

Costs of the water harvester

The total costs of water production include the operating costs and the total capital investment. Regarding the total capital investment, it consists of the total capital investment of the device and the costs of the sorbent, and each of them includes both raw materials costs and manufacturing costs.

Supplementary Table 5. Total capital investment of the device (unit: $USD)

Raw materials	Price of one prototype	Price in case of mass production	Manufacturing process	Price of one prototype	Price in case of mass production
Stainless steel	23.6	11.0	Cutting & welding	94.5	32.5
Thermal insulation foam	1.6	1.6	Assembly	7.9	7.9
Temperature-control system	34.7	28.4	Polishing	10.0	5.0
Buckles	18.9	12.6			
Total	78.8	53.6	Total	112.4	45.4

The cost assessment of the water harvester is listed in Supplementary Table 5, showing the total price of one prototype is $USD 191.2, and if it is mass-produced, the price could be reduced to less than $USD 100. The costs of the raw materials of the water harvester include the stainless steel, thermal insulation foam, buckles to seal the device, and the temperature-control system. The most expensive part is the temperature-control system ($28.4-34.7). Note that the raw materials required for the fabrication of the device are common and can be mass-produced, which further reduces the costs of the device. Besides, the plastic buckles and aluminum materials can be used to further control the costs instead of using more expensive stainless steel and metallic buckles. Regarding the manufacturing costs, the price of the manufacturing process can be divided into the depreciation costs of the equipment and the costs of labor-hour, which are more expensive than the raw materials.

Costs of the sorbent

Supplementary Table 6. The total cost of the sorbent (unit: $USD)

Raw materials	Price of one prototype	Price in case of mass production	Preparation process	Depreciation expense of the equipment	Specific energy consumption
Lithium chloride	6.4	0.3	Drying	0.04	0.42
Active carbon fiber felt	0.5	0.4	Stirring	0.03	0.16
PTFE porous membrane	0.4	0.3			
Total	7.2	1.0	Total	0.65	

The total cost of the sorbent is listed in Supplementary Table 6, and the costs of both raw materials and preparation processes were considered. The raw materials cost $USD 7.2 to prepare a piece of sorbent in the lab, and the price of LiCl accounts for 86% of the total price, because the Sigma-Aldrich or Aladdin (Chemical supplier) ACS reagent chemical was used in the lab-scale preparation (170 $/kg). However, if considering the industrialized mass production potential of LiCl, the price can be reduced by approximately 95% (8 $/kg). The industry standard chemicals can still meet the demand regarding the purity level for our applications. Furthermore, the matrix activated carbon fiber, as one kind of specially treated carbon material, can be produced by being heated from recycled organic biomass (coconut, pistachio shell, saw dust, etc.), which is cheap and sustainable. Compared to the lab-scale preparation, purchasing larger amounts (on a hundred-kilogram scale) of raw materials can further reduce the price to $USD 1.0. The results demonstrated the commercial potential and the sustainability of the sorbent.

The costs of the preparation process include the amortization expenses and the cost of labor-hour. It takes only $0.65 to prepare a piece of sorbent (625 cm^2), because the preparation of the sorbents only requires mild temperature drying and stirring dissolution, not involving expensive equipment, very high pressure/temperature conditions, and harmful processes to the human body or the environment. Overall, the cheap and industrially mass-produced raw

materials and the simple production processes makes the sorbent highly suitable for mass production and show great market potential.

To sum up, the price of 8 piece of sorbents with the required surface area that was used in the water harvester sums to $8.65 for mass production and $58.25 for lab-scale fabrication. The total capital investment includes one water harvesting device and eight pieces of sorbents, which is $249.45 for the lab prototype and $107.65 for the mass-produced device.

Operating costs

The operating costs are estimated based on the field test data. With such sorption and desorption conditions, the water harvester produced 311.69 g freshwater and consumed 0.695 kWh electricity (i.e., ~2.2 kWh/L), which is competitive compared to the active dew water collection system (~6 kWh/L in semi-arid/arid regions and ~ 1 kWh/L in humid climates)[16, 17]. Based on this demonstration, the costs of 1-liter drinking water generation are ~$ 0.19 according to the local average electricity costs. Although the costs of operation/energy consumption highly relate to the working conditions, energy supply methods (grid, PV, or the breeze electricity), and the scale of the water harvester, it still shows a potential advantage over other complex water harvesters[18, 19].

Supplementary Section 7. Details of Batch-process Operation Tests

We prepared eight sorbents for eight desorption cyclic experiments. Detailed information on these sorbents is shown in Supplementary Table 7. Briefly, the dry masses of the sorbents are 30.5-45.9 g. The masses after water sorption are in the range from 110.6 to 142.7 g. The average water uptake of these sorbents is 2.49 g/g.

Supplementary Table 7. Detailed information of sorbents used in eight desorption cyclic experiments

No.	Dry weight (g)	Sorbed water (g)	Desorbed water (g)	Collected water (g)	Desorption Ratio	Collection Ratio *
1	34.5	115.1	42.86	39.67	37.24%	92.56%
2	33.8	110.6	39.44	37.53	35.67%	95.16%
3	45.9	146.9	43.87	37.62	29.86%	85.75%
4	35.8	111.5	41.10	27.50	36.85%	66.91%
5	35.0	124.0	39.25	35.28	31.64%	89.89%
6	32.2	121.6	43.12	38.79	35.48%	89.96%
7	30.5	117.5	38.68	30.30	32.92%	78.34%
8	36.1	142.7	71.65	65.00	50.20%	90.72%

* Collection ratio: the amount of water collected divided by the amount of desorbed water.

The masses of desorbed water and collected water are also calculated. It shows that each sorbent desorbed its ~30%-50% mass after the 1-hour desorption process. The average collection ratio (~86%) demonstrated high condensation efficiency. Please note that the desorption ratio of the eighth desorption cycles seems to be higher than others, which is possibly caused by the higher desorption temperature.

During the self-controlled heating process, the heating system is not operating all time but intermittently according to the feedback of measured temperatures. As listed in Supplementary Table 8, it records the heating time during each desorption cycle, and the energy consumption was calculated by that data.

Supplementary Table 8. Detailed information related to energy consumption in eight desorption cyclic experiment

No.	Heating time	Energy consumption	Energy consumption	Accumulated energy

	(s)	(J)	(kWh)	consumption (kWh)
1	1839	342054	0.0950	0.0950
2	1768	328848	0.0913	0.1864
3	1780	331080	0.0920	0.2783
4	1471	273606	0.0760	0.3543
5	1703	316758	0.0880	0.4423
6	1703	316758	0.0880	0.5303
7	1657	308202	0.0856	0.6159
8	1528	284208	0.0789	0.6949

Supplementary Section 8. Stability Evaluation of Water Harvester

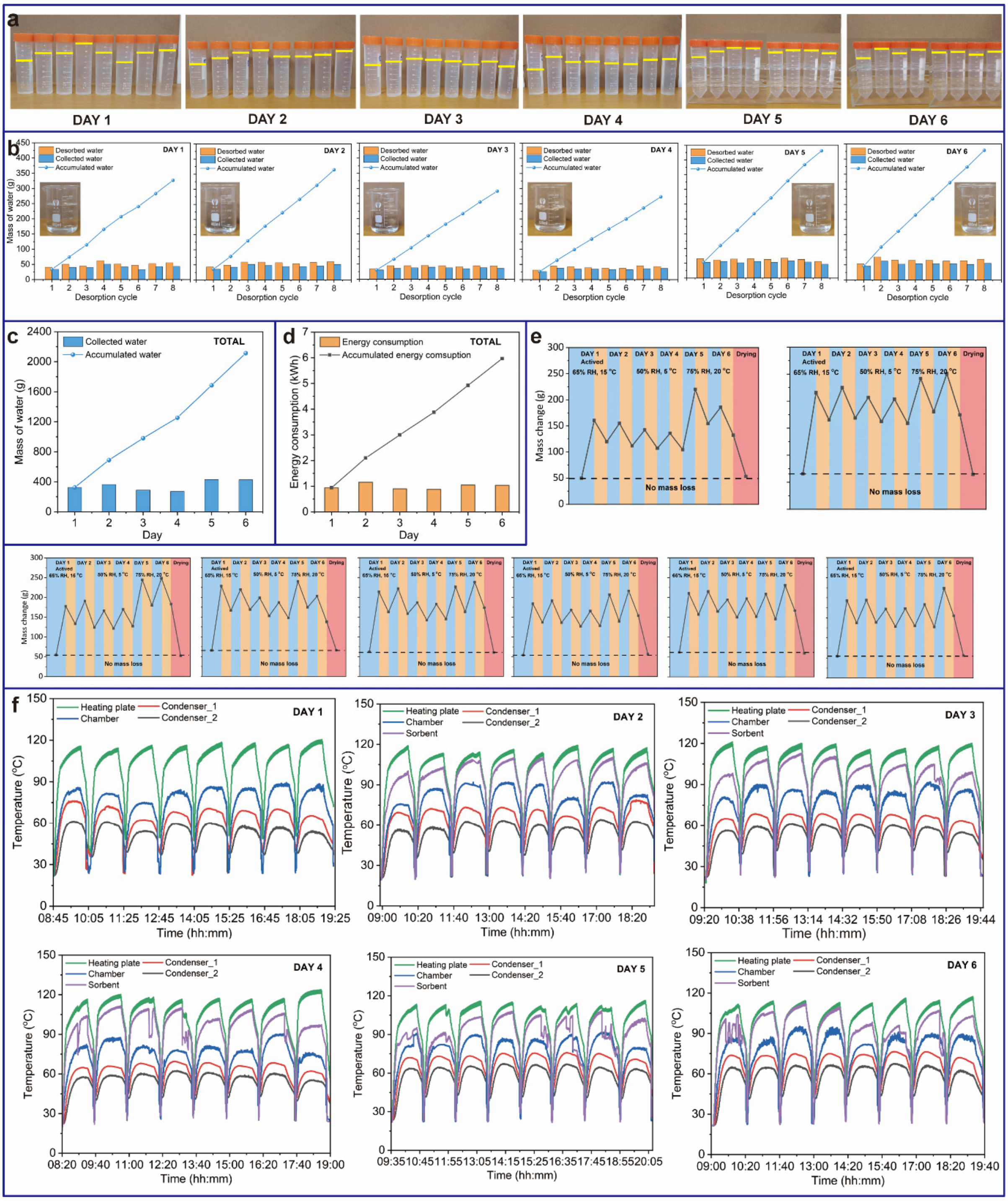

Supplementary Figure 29. Stability evaluation of the device at various simulated climate conditions. a Photos of the produced freshwater on days 1-6. **b** Collected freshwater amounts of each cycle and each day. **c** Overall water production during the cyclic tests. The inset shows the photos of the overall collected water on each day d Overall energy consumption during

the cyclic tests. **e** Mass changes of each piece of the sorbent during the 6-day water capture-release cycling tests. **f** Temperature variation of the heating plate, sorbent, chamber, and two measuring points on the condenser. The measuring points of the sorbent moved due to released vapor flow, resulting in the measured temperature fluctuations.

The three nighttime sorption conditions are the typical climate in the spring/autumn season (15°C, 65% RH), the typical climate in the winter season (5°C, 50% RH), and the typical climate in the summer season (20°C, 75% RH). Experiments under each climate condition were conducted for 2 days, with a total experimental time of 6 days. As shown in Supplementary Figure 29a-b, the water production remained consistent in each climate condition, which are ca. 340 g at the spring condition, ca. 280 g at the winter condition, and ca. 425 g in the summer condition. The total water production over the 6 days is ~2115 g (Supplementary Figure 29c), showing the excellent water production performance and the adaptivity of the water harvester to different climates. As shown in Supplementary Figure 29d, the total energy consumption is 5.97 kWh, corresponding to the water production of ~355 g per kilowatt-hour.

In addition to the overall water production and energy consumption, the mass changes of the sorbents during the cycles are shown in Supplementary Figure 29e. It shows that the average water uptake of each sorbent is 2.41 g/g, 2.04 g/g, and 2.81 g/g for each climatic condition, respectively. More importantly, all sorbents have no obvious mass loss after these cycles, showing excellent stability throughout the cyclic tests with variable working conditions. Detailed information on the temperature variation of each component of the water harvester can be found in Supplementary Figure 29f.

Supplementary Section 9. Comprehensive evaluation of portability and water production performance

The metrics (g_{water} day^{-1}, g_{water} $g_{sorbent}^{-1}$ day^{-1}, g_{water} m_{solar}^{-2} day^{-1}, g_{water} kWh^{-1}, g_{water} kg_{device}^{-1} day^{-1}, g_{water} L_{device}^{-1} day^{-1}, thermal efficiency, \$ L^{-1}) are commonly used based on different input energy sources (solar energy, PV, or low-grade energy) or various applications (portable, cyclic, vehicle-mounted, or large-scale). Among them, water productivity per solar absorbing area per day (g_{water} m_{solar}^{-2} day^{-1}) is suitable for passive solar-thermal system, while grams of water produced per unit energy consumption (g_{water} kWh^{-1}) is usually used for active systems. Here, we calculated the volume (L), weight (kg), total water production (g or mL), obtained water per day per gram of sorbent ($g_{water} \cdot g_{sorbent}^{-1} \cdot day^{-1}$), obtained water per volume ($g_{water} \cdot L_{device}^{-1} \cdot day^{-1}$) and obtained water per weight of the device ($g_{water} \cdot kg_{device}^{-1} \cdot day^{-1}$) to comprehensively evaluate the portability and the water production performance of our water harvester. Owing to the advanced thermal design of the device, the proposed multicycle operation strategy, and the high water uptake of the sorbent, our water harvester offers significant advantages in terms of portability and single-day water production performance.

Note that we included these metrics of other recently reported water harvesters. Some works report the volume and weight of devices clearly, while in some cases this data has been calculated by using similar metrics. For these works, we directly refer to its data. However, there are still many papers vaguely providing this information. We tried our best to extract the relevant information in those papers and carried out the calculations, and some related information about the devices and units could be found by searching the reported specifications and technical parameters on the internet. Unfortunately, some details still do not seem to be available in the references, hence we marked them accordingly. The metrics of some water harvesters could be rough.

Supplementary Table 9 The detailed information of calculated metrics.

Reference	Volume (L)	Weight (kg)	Obtained water (g)	$g_{water}/g_{sorbent}$/day	g_{water}/L_{device}/day	g_{water}/kg_{device}/day
This work	5.60	3.20	311.69	1.09	55.66	97.40
[20] *[1]	1086.37	N/A	38500	0.55	35.44	N/A
[18] *[2]	15.68	72.58	405.30	0.70	25.85	5.58
[21]	2.65	1.41	22.82	1.05	8.61	16.18
[22]	1.14	N/A	17.96	0.09	15.78	N/A
[23]	0.64	0.52	5.60	0.69	8.70	10.83
[24] *[3]	11.04	4.17	59.70	0.12	5.41	14.31
[25]	1.20	N/A	2.46	0.84	2.05	N/A
[26] *[4]	0.77	0.54	0.75	0.25	0.98	1.40
[27] *[5]	254.80	319.00	55.00	0.07	0.22	0.17
[28]	N/A	N/A	0.40	0.30	N/A	N/A

[1] *without the volume and weight of connection tubes, support structures, and attachments.*
[2] *without the volume and weight of PV modules, its controllers, and inverters.*
[3] *without the condensation part.*
[4] *without support structures.*
[5] *with soil insulation part.*

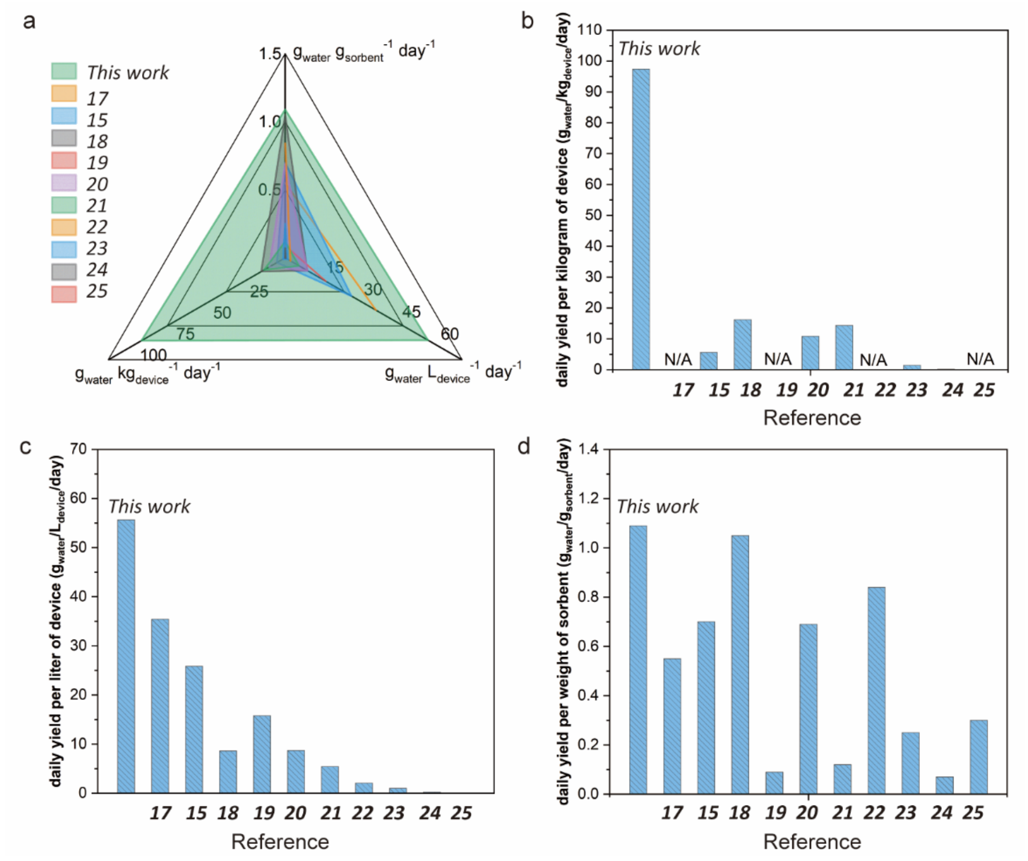

Supplementary Figure 30. Evaluation of the developed water harvester. a An evaluation of the presented water harvester in terms of comprehensively evaluated metrics based on existing sorbents and corresponding devices. **b** Daily water production yield referring to the weight of the whole device. **c** Daily water production yield referring to the volume of the whole device. **d** Daily water production yield referring to the total weight of the sorbents.

Supplementary Section 10. Freshwater production assessment

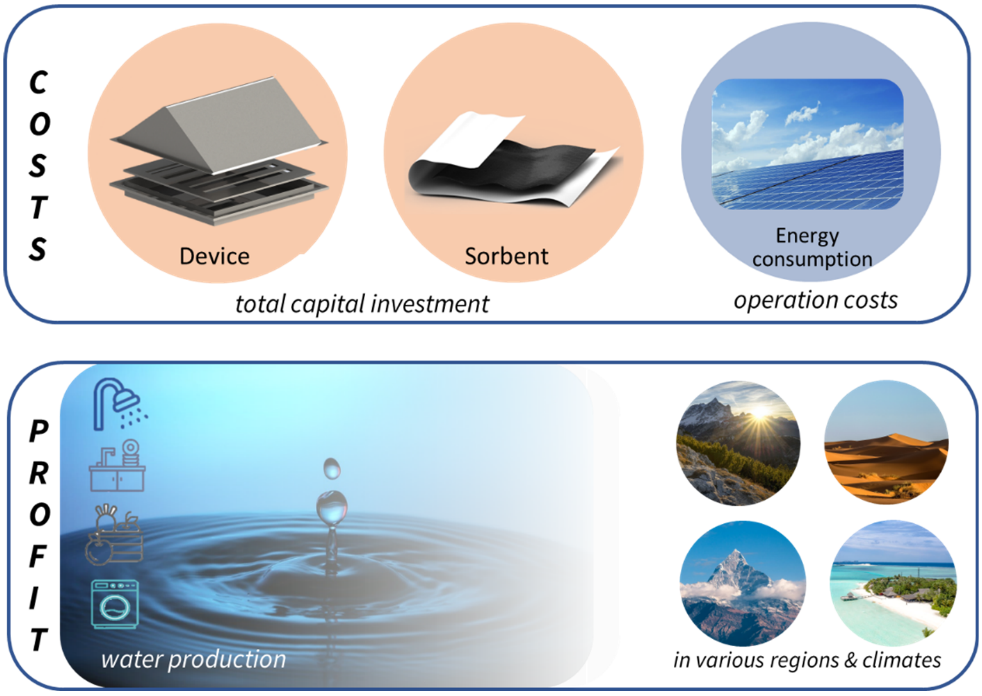

Supplementary Figure 31. Techno-economic assessment of the water harvesters with the sorbents. Before estimating the water production of the water harvester, we would like to emphasize that the minimum amount of water is ~4 L/day per person for basic personal survival, as recommended by the US National Academies of Science[29]. If considering drinking water, sanitation, bathing, and cooking, it estimated that an individual requires a minimum amount of freshwater equal to 50 L/day[30]. Researchers in this field are still pursuing to reach the goal through both materials designs and system optimizations. Beyond that, the atmospheric water harvesting technology has shown the potential to change traditional approaches to access the water, especially when it is not easily accessible such as during disasters, emergencies and in regions experiencing water stress, where the liquid water is typically carried by individuals or transported from remote locations, accompanied by heavy costs. Meanwhile, these point-of-use case use scenarios using the off-the-grid AWH devices have the potential to provide safe water by obviating the need to build pipes, use water transportation using trucks or extract contaminated surface water[31]. Therefore, as shown in Supplementary Figure 31, the techno-economic assessment we conducted is referring to the AWH technology that can provide safe freshwater anytime and anywhere.

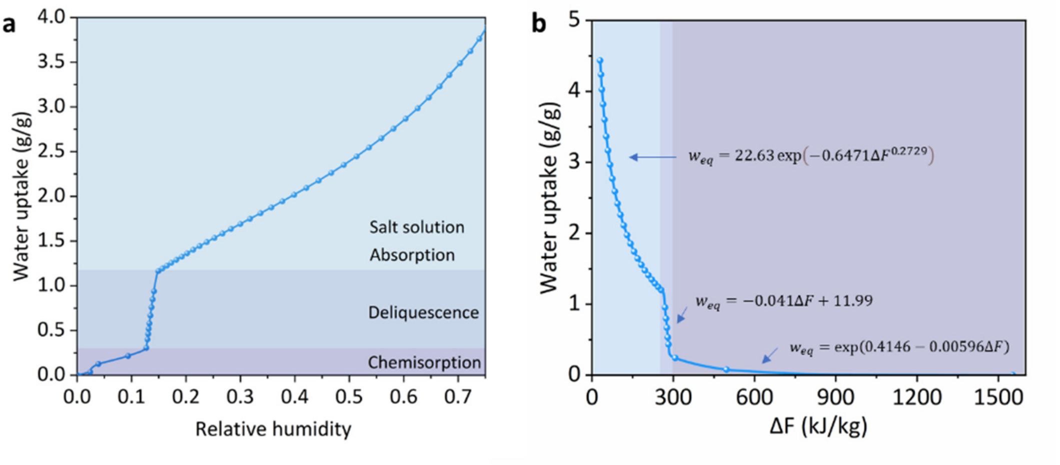

Supplementary Figure 32. Prediction of the sorption amount during the daytime under various conditions. a Experiment vapor sorption isotherm at 30 °C**. b** Calculated D-A fitting equation curve.

We estimate the water production of the water harvester at two levels. One is the global annual average water production, and the other is water production estimation during different seasons in five typical climates. Due to the variability of the sorption temperature and RH, we firstly established a model for predicting the water sorption amount of the sorbents under various sorption conditions. The Dubibib-Astakhov (D-A) equation was selected to predict the sorption capacity, which is derived from the experimental vapor sorption isotherm (Supplementary Figure 32a).[32] The equation can be expressed as which can be express as:

$$w_{eq} = w_0 \exp(-k\Delta F^n)$$

where w_{eq} is the equilibrium sorption capacity (g/g), and w_0, k, n are the fitting coefficients. In the above equation, ΔF is the free sorption energy (i.e, sorption potential), which is the function of relative pressure (relative humidity) and the temperature:

$$\Delta F = -RT\ln\left(\frac{P_v}{P_{sat}}\right)$$

where R is the gas constant (J·mol^{-1}·K^{-1}), P_v, P_{sat} are the partial pressure and the saturated vapor pressure of water vapor (Pa) respectively. Therefore, various sorption condition (temperature and RH) at different locations and seasons can be expressed by only one parameter – the free sorption energy. The fitting equation results are shown in Supplementary Figure 32b and listed in Supplementary Table 10.

Supplementary Table 10 D-A sorption characteristic fitting curve of the sorbent

Stage	F (kJ/kg)	Correlation curves	R^2
I	1552.77-285.69	$w_{eq} = \exp(0.4146 - 0.00596\Delta F)$	0.9779
II	285.69-264.24	$w_{eq} = -0.041\Delta F + 11.99$	0.9907
III	264.24-29.49	$w_{eq} = 22.63\exp(-0.6471\Delta F^{0.2729})$	0.9996

After that, the global average annual temperature and RH data were obtained from Univ. of East Anglia and used together with the above equations to estimate the harvested water amount of the sorbent[33]. Then, the average water production is calculated based on the assumption of constant desorption and condensation rate that we obtained from the field tests. Meanwhile, due to the dispersion and nonuniformity of weather stations, a two-dimensional grid scatter interpolation method based on one-dimensional periodic boundary conditions of longitude is used for interpolation. A map toolbox from MATLAB software was used to represent the water production map.

The estimated daily water production of the water harvester working in the batch-process mode is shown in Fig. 5b. The results show that the global average annual water production is around 500 mL. The water harvester works in the Sahara Desert with ultra-low RH, yet it could still produce an average of 200 mL freshwater per day, showing excellent water sorption capacity of the sorbents and efficient freshwater production by the water harvester.

To be more specific, we selected five global climates globally with their corresponding monthly average ambient conditions (temperature and RH) which were obtained from the Energy Plus software, as shown in Supplementary Figure 33a-f. The five regions listing five typical climates were Birmingham, UK (humid climate and low ambient temperature), Chennai, India (humid climate and high ambient temperature), Kharga, Egypt (arid climate, near the Sahara Desert), Barstow, California (arid climate with high ambient temperature, near the Mojave Desert),

Lanzhou, China (semi-arid climate with large seasonal temperature differences). Different from the global water production estimation using the yearly average RHs, the water production for the five typical climates used the nighttime temperature and RHs (from 18:00 to 06:00 the next day), which improves the estimation accuracy. Besides, as shown in Supplementary Figure 33a-e, we selected two typical months with the lowest or highest monthly average RHs in the whole year in each region. We believe these can further comprehensively evaluate the working performance of the water harvester in both different locations and seasons.

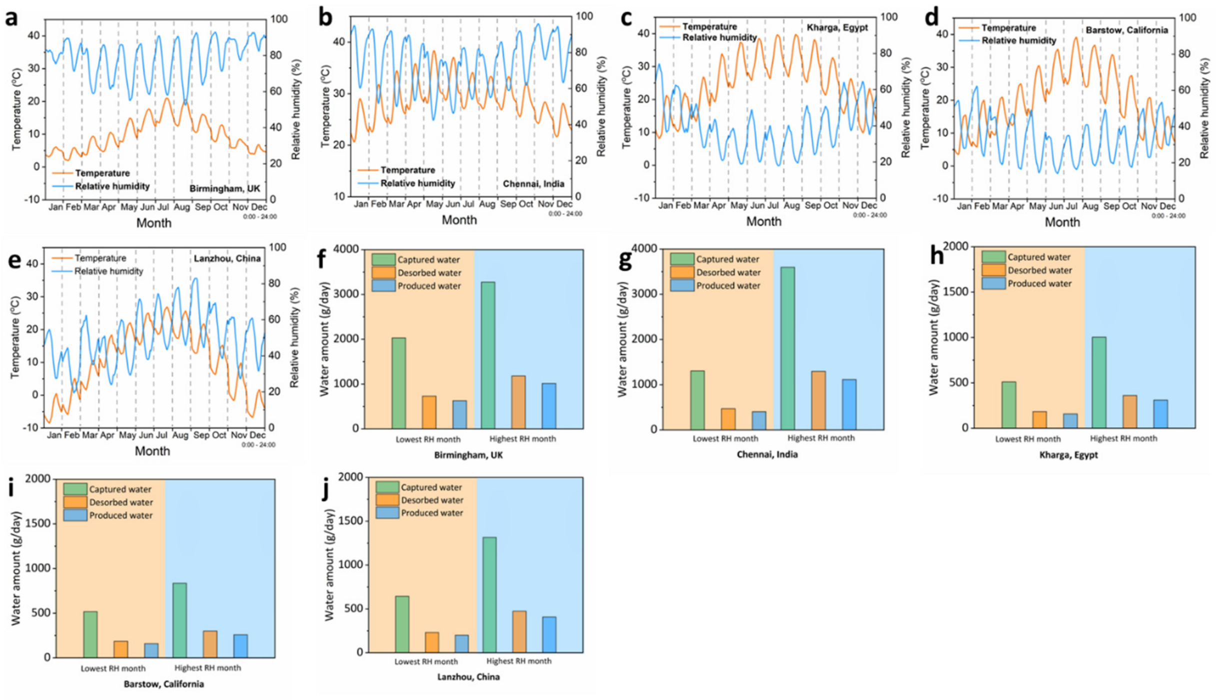

Supplementary Figure 33. Monthly average typical daily temperature and relative humidity. **a** Birmingham, UK; **b** Chennai, India; **c** Kharga, Egypt; **d** Barstow, California; **e** Lanzhou, China. Predicted daily water production in **f** Birmingham, UK; **g** Chennai, India; **h** Kharga, Egypt; **i** Barstow, California; **j** Lanzhou, China

The predicted water production (g/day) of each location and climate is shown in Supplementary Figure 33g-l. The results show that the production is significantly influenced by the ambient relative humidity. For instance, the production in relative humid regions (such

as, Birmingham, UK and Chennai, India) was calculated to be over 1,000 g per day in high RH months. In contrast, the minimum water productions that can be achieved in the low RH reasons in desert arid climates (Kharga, Egypt and Barstow, California) was estimated at ca. 160 g per day, which could be considered as the minimum daily water production of the water harvester. All the water production estimations demonstrated the adaptivity and excellent working performance of the water harvester.

Supplementary Section 11. Estimation of PV-powered Water Harvesting

The portable sorption-based water harvester could be carried for water harvesting anywhere, therefore, given the possibility of the lack of commercial power grid, and considering the high correspondence between arid regions and solar-rich regions, solar PV system was considered as an alternative method to power the portable device. The monthly average sunshine hours in Lanzhou were provided by Meteorological Data Center of China Meteorological Administration (Supplementary Figure 34).

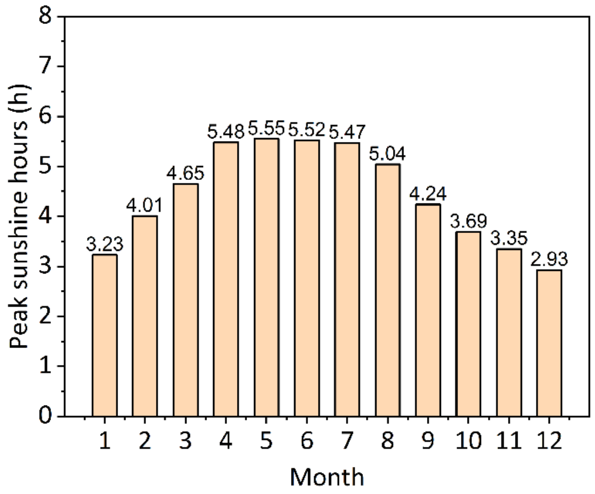

Supplementary Figure 34. Average peak sunshine hours in Lanzhou, China

The heating power of the device was 186 W, and the calculation method refer to the data given by commercial PV companies. Firstly, we designed the total capacity of solar cell module:

$$P = A\frac{P_H}{T_H} = (1.5{\sim}2.0)\frac{P_H}{T_m}$$

P – Calculated solar cell module power.

A – Overall coefficient (1.5-2.0), which includes the influences of the following parameters:

- Installation angle
- Temperature losses
- Cables losses
- Shadings
- Losses at weak radiation

- Losses due to dust, snow...

- Other Losses

T_m – Average peak sunshine hours (Supplementary Figure 34).

P_H – Load power, which is 186 W in this case.

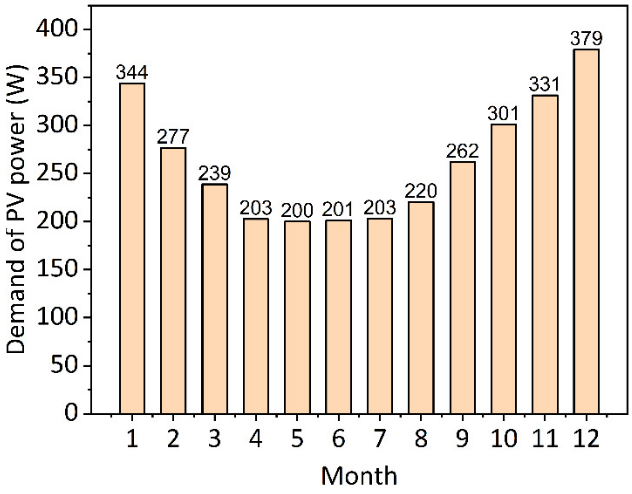

Supplementary Figure 35. Calculated solar cell module power in different months.

Therefore, according to the local monthly average peak sunshine hours (5.55-2.93 h) and the overall coefficient (1.6)[34], the calculated demand for solar cell modules is 200-378 W (Supplementary Figure 35).

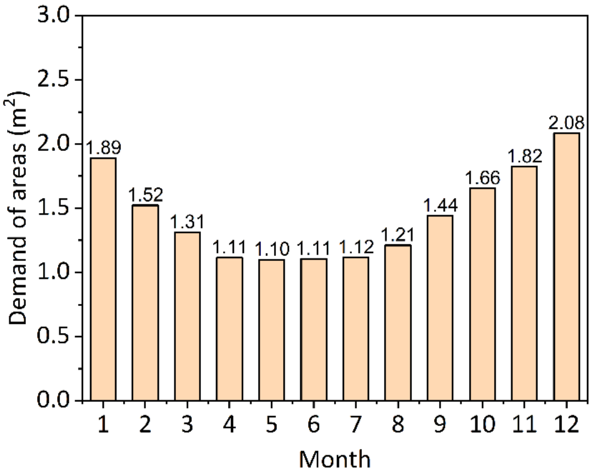

Supplementary Figure 36. Required areas of solar modules in different months

The minimum solar cell power is 200 W. Commercial 200 W monocrystalline solar panels with an area of 1.1 m^2 can meet this demand, which is made by Grape Solar (https://grapesolar.com/). According to the data of this solar panel, we further calculated the required areas of the solar modules (Supplementary Figure 36).

In the actual situation, considering the irradiation intensity variations that is influenced by weather (even considering rainy days), a 12 V battery system becomes necessary to store the remaining electricity during sunny days. The required battery capacity was calculated by[34, 35]:

$$C_{bat} = \frac{P_d}{V}\frac{1}{N_d \eta}$$

P_d – Daily energy consumption ($P_d = 0.694$ kWh, according to the 8 cyclic experiment)
V – Voltage of the heating plate (12 V)
η – System overall efficiency (0.85)
N_d - Depth of discharge (0.8)

The calculated battery capacity is 102 Ah (12V). In the case of several days with continuous rain, this capacity needs to be increased.

Supplementary Table 14 The required solar PV power and PV areas

Month	Peak sunshine hours (h)	Efficiency factor	Required PV power (W)	Energy consumption per day (Wh)	Required PV area(m^2)
Jan.	3.23	1.60	343.78	694.00	1.89
Feb.	4.01	1.60	276.91	694.00	1.52
Mar.	4.65	1.60	238.80	694.00	1.31
Apr.	5.48	1.60	202.63	694.00	1.11
May	5.55	1.60	200.07	694.00	1.10
Jun.	5.52	1.60	201.16	694.00	1.11
Jul.	5.47	1.60	203.00	694.00	1.12
Aug.	5.04	1.60	220.32	694.00	1.21

Sep.	4.24	1.60	261.89	694.00	1.44
Oct.	3.69	1.60	300.92	694.00	1.66
Nov.	3.35	1.60	331.46	694.00	1.82
Dec.	2.93	1.60	378.98	694.00	2.08

To sum up, the basic requirement of running the device over eight cycles per day requires the power of solar cell modules in the range of 200-378 W and the module area in the range of 1.11-2.08 m^2. The required battery capacity is 102 Ah.

References

1. Yan S*, et al.* Effect of fiber content on the microstructure and mechanical properties of carbon fiber felt reinforced geopolymer composites. *Ceramics International* **42**, 7837-7843 (2016).

2. He P*, et al.* Effects of fiber contents on the mechanical and microwave absorbent properties of carbon fiber felt reinforced geopolymer composites. *Ceramics International* **44**, 10726-10734 (2018).

3. Lin T, Jia D, He P, Wang M. In situ crack growth observation and fracture behavior of short carbon fiber reinforced geopolymer matrix composites. *Materials Science and Engineering: A* **527**, 2404-2407 (2010).

4. Lawson KW, Lloyd DR. Membrane distillation. *Journal of Membrane Science* **124**, 1-25 (1997).

5. Wang W*, et al.* Integrated solar-driven PV cooling and seawater desalination with zero liquid discharge. *Joule* **5**, 1873-1887 (2021).

6. Baghel R, Upadhyaya S, Singh K, Chaurasia SP, Gupta AB, Dohare RK. A review on membrane applications and transport mechanisms in vacuum membrane distillation. *Reviews in Chemical Engineering* **34**, 73-106 (2018).

7. Alkhudhiri A, Darwish N, Hilal N. Membrane distillation: A comprehensive review. *Desalination* **287**, 2-18 (2012).

8. Sircar S. Linear-driving-force model for non-isothermal gas adsorption kinetics. *Journal of the Chemical Society, Faraday Transactions 1: Physical Chemistry in Condensed Phases* **79**, 785-796 (1983).

9. Sircar S, Hufton JR. Why Does the Linear Driving Force Model for Adsorption Kinetics Work? *Adsorption* **6**, 137-147 (2000).

10. Peeters R, Verbruggen V, Rongé J, Martens JA. Non-Isothermal Kinetic Model of Water Vapor Adsorption on a Desiccant Bed for Harvesting Water from Atmospheric Air. *Industrial & Engineering Chemistry Research* **60**, 11812-11823 (2021).

11. Legrand U, Girard-Lauriault P-L, Meunier J-L, Boudreault R, Tavares JR. Experimental and Theoretical Assessment of Water Sorbent Kinetics. *Langmuir* **38**, 2651-2659 (2022).

12. Díaz-Marín CD, Zhang L, Lu Z, Alshrah M, Grossman JC, Wang EN. Kinetics of Sorption in Hygroscopic Hydrogels. *Nano Letters* **22**, 1100-1107 (2022).

13. Roy PK, Legchenkova I, Shoval S, Bormashenko E. Interfacial Crystallization within Janus Saline Marbles. *The Journal of Physical Chemistry C* **125**, 1414-1420 (2021).

14. Markatos NC, Pericleous KA. Laminar and turbulent natural convection in an enclosed cavity. *International Journal of Heat and Mass Transfer* **27**, 755-772 (1984).

15. Jagirdar M, Lee PS. Mathematical modeling and performance evaluation of a desiccant coated fin-tube heat exchanger. *Applied Energy* **212**, 401-415 (2018).

16. Wahlgren RV. Atmospheric water vapour processor designs for potable water production: a review. *Water Research* **35**, 1-22 (2001).

17. Tu Y, Wang R, Zhang Y, Wang J. Progress and Expectation of Atmospheric Water Harvesting. *Joule* **2**, 1452-1475 (2018).

18. Hanikel N*, et al.* Rapid Cycling and Exceptional Yield in a Metal-Organic Framework Water Harvester. *ACS Cent Sci* **5**, 1699-1706 (2019).

19. Wang W, Xie S, Pan Q, Dai Y, Wang R, Ge T. Air-cooled adsorption-based device for harvesting water from island air. *Renewable and Sustainable Energy Reviews* **141**, (2021).

20. Wang JY, Wang RZ, Tu YD, Wang LW. Universal scalable sorption-based atmosphere water harvesting. *Energy* **165**, 387-395 (2018).

21. Xu J*, et al.* Ultrahigh solar-driven atmospheric water production enabled by scalable rapid-cycling water harvester with vertically aligned nanocomposite sorbent. *Energy & Environmental Science*, (2021).

22. Qi H*, et al.* An Interfacial Solar‐Driven Atmospheric Water Generator Based on a Liquid Sorbent with Simultaneous Adsorption–Desorption. *Advanced Materials* **31**, (2019).

23. Shan H*, et al.* High-yield solar-driven atmospheric water harvesting with ultra-high salt content composites encapsulated in porous membrane. *Cell Reports Physical Science*, (2021).

24. LaPotin A*, et al.* Dual-Stage Atmospheric Water Harvesting Device for Scalable Solar-Driven Water Production. *Joule* **5**, 166-182 (2021).

25. Li R, Shi Y, Wu M, Hong S, Wang P. Improving atmospheric water production yield: Enabling multiple water harvesting cycles with nano sorbent. *Nano Energy* **67**, (2020).

26. Kim H*, et al.* Adsorption-based atmospheric water harvesting device for arid climates. *Nat Commun* **9**, 1191 (2018).

27. Fathieh F, Kalmutzki MJ, Kapustin EA, Waller PJ, Yang J, Yaghi OM. Practical water production from desert air. *Science Advances* **4**, eaat3198 (2018).

28. Kim H*, et al.* Water harvesting from air with metal-organic frameworks powered by natural sunlight. *Science* **356**, 430-434 (2017).

29. Meyers LD, Hellwig JP, Otten JJ. *Dietary reference intakes: the essential guide to nutrient requirements*. National Academies Press (2006).

30. Gleick PH. Basic Water Requirements for Human Activities: Meeting Basic Needs. *Water International* **21**, 83-92 (1996).

31. Humphrey JH*, et al.* The potential for atmospheric water harvesting to accelerate household access to safe water. *The Lancet Planetary Health* **4**, e91-e92 (2020).

32. Stoeckli HF, Kraehenbuehl F, Ballerini L, De Bernardini S. Recent developments in the Dubinin equation. *Carbon* **27**, 125-128 (1989).

33. https://sage.nelson.wisc.edu/data-and-models/atlas-of-the-biosphere/mapping-the-biosphere/ecosystems/average-annual-relative-humidity/. *Climate Research Unit, Univ of East Anglia*, (1999).

34. Mahmoud MM, Ibrik IH. Techno-economic feasibility of energy supply of remote villages in Palestine by PV-systems, diesel generators and electric grid. *Renewable and Sustainable Energy Reviews* **10**, 128-138 (2006).

35. Aglietti GS, Redi S, Tatnall AR, Markvart T. Harnessing High-Altitude Solar Power. *IEEE Transactions on Energy Conversion* **24**, 442-451 (2009).
